# Cerebellum Lecture: the Cerebellar Nuclei—Core of the Cerebellum

**DOI:** 10.1007/s12311-022-01506-0

**Published:** 2023-02-13

**Authors:** Justus M. Kebschull, Filippo Casoni, G. Giacomo Consalez, Daniel Goldowitz, Richard Hawkes, Tom J. H. Ruigrok, Karl Schilling, Richard Wingate, Joshua Wu, Joanna Yeung, Marylka Yoe Uusisaari

**Affiliations:** 1 Department of Biomedical Engineering, Johns Hopkins University, Baltimore, MD 21205 USA; 2 Division of Neuroscience, San Raffaele Scientific Institute, and San Raffaele University, Milan, Italy; 3 Department of Medical Genetics, Centre for Molecular Medicine and Therapeutics, University of British Columbia, Vancouver, Canada; 4 Department of Cell Biology & Anatomy and Hotchkiss Brain Institute, Cumming School of Medicine, University of Calgary, Calgary, Alberta T2N 4N1 Canada; 5 Department of Neuroscience, Erasmus MC, Rotterdam, the Netherlands; 6 Department of Anatomy, Anatomy & Cell Biology, Rheinische Friedrich-Wilhelms-Universität, 53115 Bonn, Federal Republic of Germany; 7 MRC Centre for Neurodevelopmental Disorders, Institute of Psychiatry, Psychology and Neuroscience, King’s College London, London, UK; 8 Neuronal Rhythms in Movement Unit, Okinawa Institute of Science and Technology, 1919-1 Tancha, Onna-Son, Kunigami-Gun, Okinawa 904-0495 Japan

**Keywords:** Cerebellum, Cerebellar nuclear afferents, Cerebellar nuclear efferents, Cerebellar nuclear anatomy, Cerebellar nuclear cell types, Cerebellar modules, Development, Rhombic lip, Ventricular zone, Evolution, Molecular specification, Neurogenesis, Migration, Pathology, Cerebellar ataxias, Connectivity, Mouse, Chick

## Abstract

The cerebellum is a key player in many brain functions and a major topic of neuroscience research. However, the cerebellar nuclei (CN), the main output structures of the cerebellum, are often overlooked. This neglect is because research on the cerebellum typically focuses on the cortex and tends to treat the CN as relatively simple output nuclei conveying an inverted signal from the cerebellar cortex to the rest of the brain. In this review, by adopting a nucleocentric perspective we aim to rectify this impression. First, we describe CN anatomy and modularity and comprehensively integrate CN architecture with its highly organized but complex afferent and efferent connectivity. This is followed by a novel classification of the specific neuronal classes the CN comprise and speculate on the implications of CN structure and physiology for our understanding of adult cerebellar function. Based on this thorough review of the adult literature we provide a comprehensive overview of CN embryonic development and, by comparing cerebellar structures in various chordate clades, propose an interpretation of CN evolution. Despite their critical importance in cerebellar function, from a clinical perspective intriguingly few, if any, neurological disorders appear to primarily affect the CN. To highlight this curious anomaly, and encourage future nucleocentric interpretations, we build on our review to provide a brief overview of the various syndromes in which the CN are currently implicated. Finally, we summarize the specific perspectives that a nucleocentric view of the cerebellum brings, move major outstanding issues in CN biology to the limelight, and provide a roadmap to the key questions that need to be answered in order to create a comprehensive integrated model of CN structure, function, development, and evolution.

## Introduction

*The cerebellum has unquestionably given more trouble to anatomists than any other organ, and our knowledge of its structure seems disproportionate to the labor expended.*

*C. L. Herrick (1891). The evolution of the cerebellum. Science* ***18****: 188-189.*

The above quotation seems to have lost little of its significance in the 130 years that have passed since Herrick’s frustrations. Although initially the cerebellum was seen as a system involved in controlling and coordinating movements [e.g., 1], it has now become evident that memory processes required for associative learning and adapting of motor functions are also controlled by the cerebellum [2, 3]; and more recently the cerebellum has been shown to be involved in autonomic, emotional and cognitive aspects of brain function [4, 5, 6, –8].

While much clarity has emerged concerning the cerebellar cortex, the same cannot be said of the cerebellar nuclei (CN). However, hardly any of the cerebellar cortical computations reach the rest of the brain nor impact behavior unless they are processed by the CN. Almost all cerebellar output is in fact constructed within the circuits of the CN, by means of integrating a wide range of inputs from the entire CNS with the modulatory influences of cerebellar cortical afferents. We therefore posit that understanding the CN is essential to understanding the cerebellum. Here, we present a comprehensive review of the structure, physiology, development, and evolution of the CN.

**Fig. 1:**
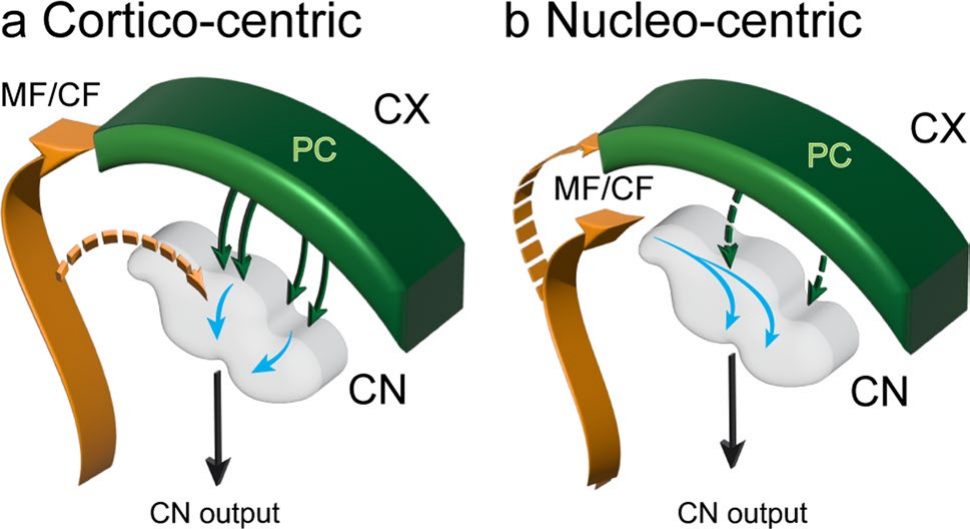
Panel **a** shows the conventional, cortico-centric, model by which the cerebellar circuitry is described. Afferent inputs, conveyed by CF (climbing fibers) and MF (mossy fibers) terminate predominantly in the cerebellar cortex, with collateral copies to the CN, which are thought to be of lesser importance. The cerebellar cortex processes the signal, which then passes from the Purkinje cells (PC) to the cerebellar nuclei (CN) and out of the cerebellum. Panel **b** illustrates the nucleo-centric perspective. The primary pathway is for cerebellar afferents to synapse in the CN, where the cerebellar efferents originate. In parallel, afferent copies are sent to the cerebellar cortex, where a complementary inhibitory signal is generated that enters the CN via the corticonuclear pathway and modulates the cerebellar efferent output. Please note that the thickness of the arrows reflects the relative importance of the information flow, not anatomical size or signal strength. Furthermore, blue arrows denote information passing through the CN circuits, without reference to specific cell types

Figure 1 contrasts two strategic perspectives of the cerebellum. Figure 1a illustrates the conventional perspective with a focus on the cerebellar cortex. A wide array of afferent projections—typified here by the mossy and climbing fibers (MF and CF)—innervate the cerebellar cortex, with minor collateral projections to the CN. In the cerebellar cortex the afferent information undergoes extensive computational processing and then is projected via the Purkinje cells (PC) to the CN, which distribute it widely via their efferent outputs. Figure 1b shows the same circuitry but with a nucleocentric emphasis—the projections to the CN are viewed as the primary ones, while the cerebellar cortical afferents are seen to be the secondary branches (also see “Extracerebellar Nuclear Afferents: Primary Branches or Collaterals of Cerebellar Cortical Afferents?”). The primary flow of information through the cerebellum is from cerebellar afferents to the CN and the CN efferents, with the corticonuclear input from the cerebellar cortex largely modulatory. Although there is no qualitative difference between models A and B, we propose that viewing the cerebellar system from a nucleocentric perspective will reveal novel insights into the mechanisms underlying its organization and development as well as computational function.

## Morphology and Connectivity

### History and Notes on Nomenclature

“What exactly constitutes the canonical CN?” is a valid question that could be addressed morphologically, hodologically, or developmentally. Here, for practical reasons, we wish to adhere to the classical definition that, as indicated by their name, the CN fully reside within the cerebellum and as such constitute an integral part of the cerebellum. Of course, seen from the nucleocentric perspective as taken in this review, neurons in, e.g., the vestibular and parabrachial nuclei that receive cerebellar cortical input (i.e., from PCs) may take up a similar anatomical-functional position as the neurons of the CN [9–11]. However, as these neurons will also be more intricately integrated within neuronal groups that do not receive direct Purkinje cell input, and moreover, it is uncertain to what extent their developmental and evolutionary origins are similar to those of the CN, we refrain from dealing with neuronal groups outside of the classically defined CN. The only exception will be made for the lateral vestibular nucleus (or Deiters’ nucleus) when describing the modular arrangement of olivo-cortico-nuclear connections (see **“**Cerebellar Modules”). Especially, the dorsal part of Deiters’ nucleus, forming a neuronal connection between the roof and the floor of the 4th ventricle, resembles more a cerebellar than a vestibular nucleus from both a cytological as well as a connectivity point of view [12]. Yet, because comprehensive reviewing of this and other extracerebellar sources of cerebellar cortical input will distract from the main purpose of this review, we will further confine ourselves by using a strict sense of the CN.

A first description of the CN as a nuclear mass separated from the cerebellar cortex by the cerebellar white matter was provided by Vieussens [13]. The striking appearance of its main mass as a “toothed” nucleus was noted late in the eighteenth century by Vicq-d’Azyr [in 14]. Stilling [15] provided the first description of the human CN as comprising four separate nuclei which he named the fastigial (due to its location next to the apex of the 4th ventricle), globose (ball-like), emboliform (plug-shaped), and dentate nucleus. Weidenreich [16] recognized similar nuclear groups (or nuclear complexes) in various mammals and noted that the mediocaudal complex of fastigial/globose nuclei is somewhat separated from the rostrolateral complex consisting of the emboliform/dentate nuclei. This anatomical separation, however, is less clear in the rodent. Ogawa [17], in his study on aquatic mammals, used the terms anterior interposed (IntA) and posterior interposed (IntP) nuclei for the emboliform and globose nuclei, respectively. In this review, we will adopt the terminology advocated by Paxinos and Watson [18] who also use the same terms for IntA and IntP, but refer to the fastigial and dentate nuclei as the medial (Med) and lateral (Lat) CN respectively. In referring to subregions (i.e., subnuclei) of these nuclei, we follow Korneliussen’s nomenclature [19]. The term “CN subdivision” will be used to indicate a presently less well-detailed part or cluster of the CN. Table 1 provides an overview of the terminology.

**Table 1:** Nomenclature of the canonical cerebellar nuclear complex

Cerebellar nuclei in mouse	Abbrev	Latin name	Human/primate (Eng./Latin)
Medial cerebellar nucleus	Med	Nucleus medialis cerebelli	Fastigial nucleus/nucleus fastigii
Rostral part**	rMed		
Ventral part**	vMed		
Caudal part**	cMed		
Dorsolateral protuberance*	MedDL		
Rostral dorsolateral protuberance*	MedrDL		
Caudal dorsolateral protuberance*	MedcDL		
Posterior interposed nucleus	IntP	Nucleus interpositus posterior	globose nucleus/nucleus globosus
Interstitial cell groups*	IntIC		
Anterior interposed nucleus	IntA	Nucleus interpositus anterior	emboliform nucleus/nucleus emboliformis
Dorsomedial crest*	IntDM		
Dorsolateral hump*	IntDL		
Lateral cerebellar nucleus	Lat	Nucleus lateralis cerebelli	dentate nucleus/nucleus dentatus
Ventral/parvicellular part	vLat		Macrogyric part
Dorsal/magnocelluar part	dLat		Microgyric part

Subnuclei (indicated *) are based on Korneliussen [19]; subdivisions of Med (indicated **) are based on Fujita *et al*. [20]. The names of human nuclei follow Weidenreich [16]

**Fig. 2:**
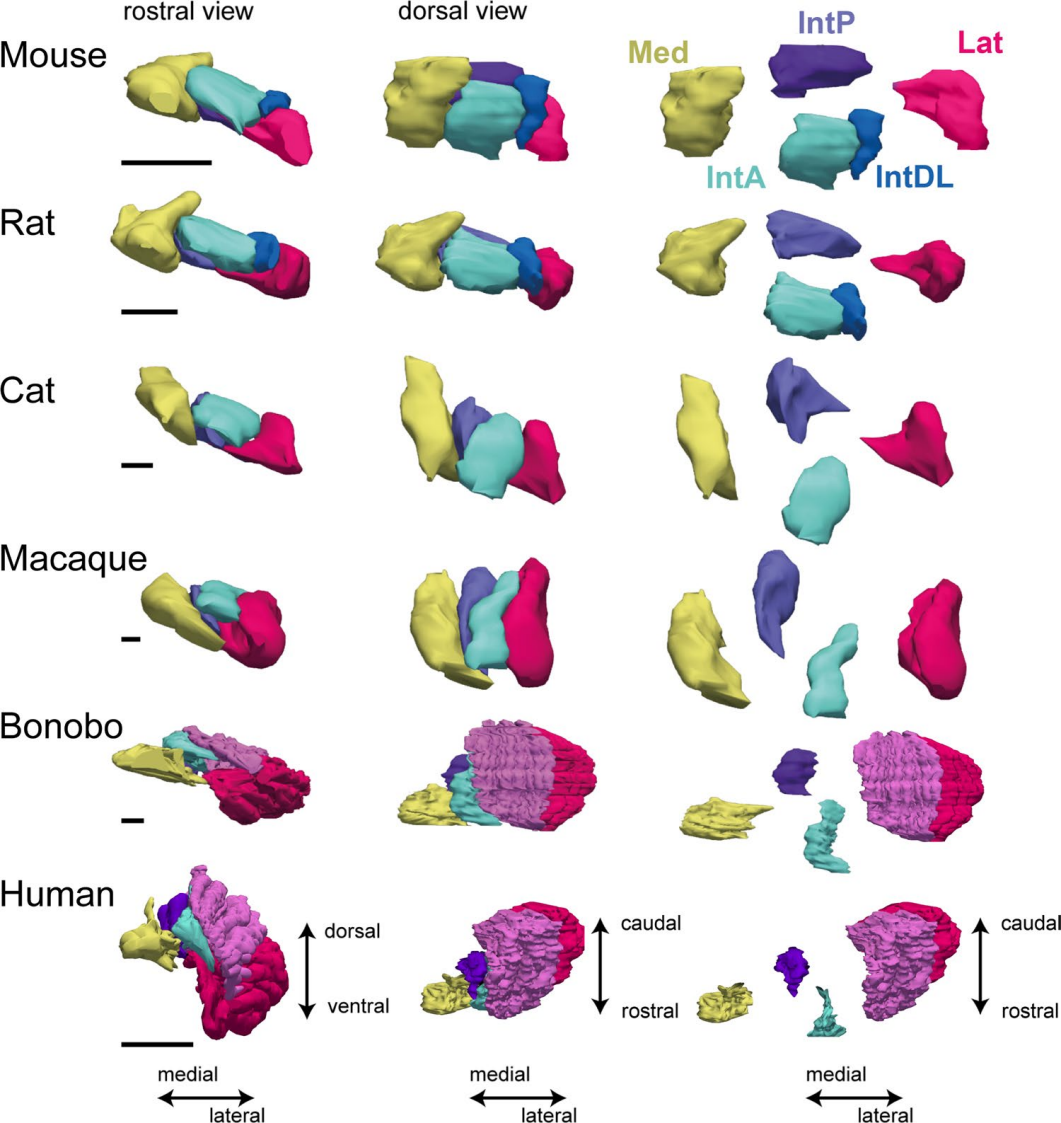
3D-reconstructions based on serial sections of the CN of the mouse, rat, cat, macaque, bonobo and human. Each horizontal panel depicts a rostral (anterior) view, a dorsal view, and a dorsal view with separated individual nuclei. Note that the relative size and shape of the various nuclei can vary considerably. The dentated appearance of the Lat can only be appreciated in the bonobo and human. In apes and humans, the gyration of the Lat can be divided into a caudoventral macrogyric (red) and rostrodorsal microgyric (purple) part. These dentated sheets of cells fold over the hilus that, in a rostral and medioventral direction, gives access to the scp. Scale bars indicate 1 mm (mouse, rat, cat and macaque), 2 mm (bonobo) and 10 mm (human). Reconstructions were made with Neurolucida (MBF Bioscience)

To illustrate the diversity of sizes and shapes of the CN of different mammals, reconstructions of the CN complex of rodents (mouse and rat), the cat, and various primates are shown in Fig. 2. Within the cerebellum as a whole, there is intriguing evidence of mosaic evolution coupled to cerebellar function and the lifestyle of the species. Within the cerebellar cortex, the 4 transverse zones (see **“**Cerebellar Modules”) seem to have evolved as independent units. Thus, for example, in most mammals the central zone (~ lobules VI–VII) is a strong recipient of visual inputs. Notably, in species in which vision has been lost, e.g., in moles, this central zone is atypically small [21, 22]. Conversely, in microchiropteran bats, in which the central zone is the recipient of massive echolocation sensory input, the central zone is unusually large [23]. The same mosaic evolution may apply also to the CN as their relative sizes, their related afferent sources, and efferent targets vary considerably across species. Thus, the different relative sizes of the individual CN may very well correlate with lifestyle, or at least be proportional to the size of the cerebellar cortical areas that innervate them. For instance, in the great apes, including humans, the Lat shows the most conspicuous relative growth in volume resulting in its remarkable dentated morphology [24]. On the other hand, in cetaceans (whales, dolphins) the IntP has expanded enormously, while maintaining its globular appearance [25, 26]. Although these examples of differential evolution are clearly linked to a similar increase in the sizes of related parts of the cerebellum, their functional meaning is still widely discussed [27–29].

### Morphology of the Murine CN Complex

Currently, mice are the most widely used experimental animals in cerebellar research. Hence, we will here review the mouse CN complex in detail and distinct features of CN in other species will be discussed only in passing.

The IntA encompasses the largest volume of the CN in mice and is followed in size by the Med and Lat. The IntP, at about half the size of the Med, constitutes the smallest nucleus. The total number of CN neurons in one-half of the mouse cerebellum has been estimated at approximately 20,000 [30]. As already mentioned, it should be noted that absolute and relative nuclear sizes as well as cell numbers vary greatly even between mammalian species (Fig. 2; thorough comparative studies are lacking for non-mammalian species). A comprehensive overview of the various cell types of the CN is given later (“Cell Types of the Adult Cerebellar Nuclei”).

#### Medial cerebellar nucleus

**Fig. 3:**
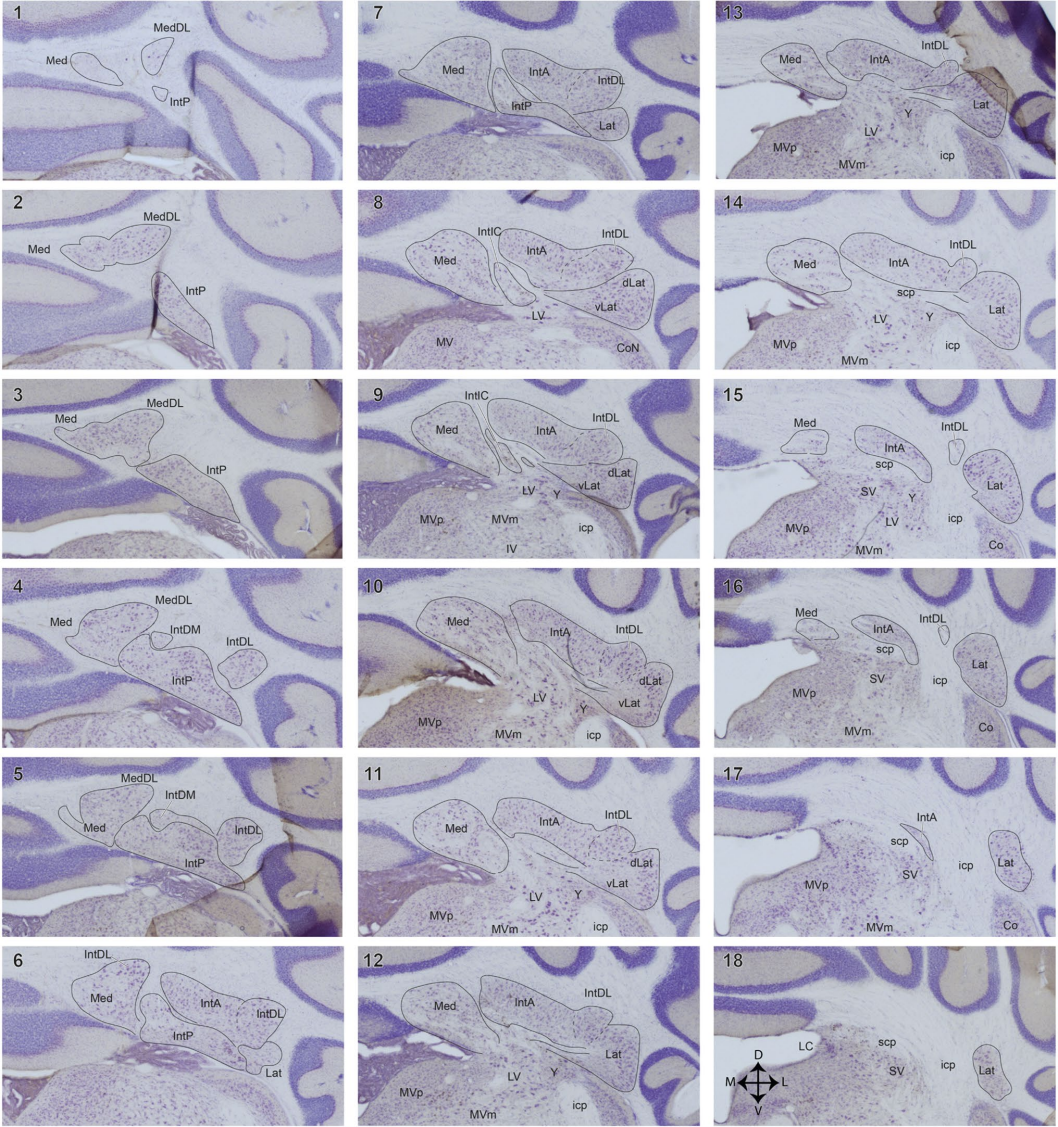
Series of equidistant (80 µm) photomicrographs of transverse, thionine-stained Sects. (40 µm) of the mouse CN from its caudal-most level (panel 1) to its rostral-most level (panel 18). Midline is at the left-hand margin of each panel. The four main nuclei are indicated by thin lines. Dashed lines indicate equivocal nuclear borders. Arrows in panel 18 denote medial (M), dorsal (D), lateral (L), and ventral (V) directions. Scale bar in panel 1 equals 500 µm. Abbreviations: CoN, cochlear nuclei; dLat, dorsal part of the Lat; icp, inferior cerebellar peduncle; IntA, anterior cerebellar nucleus; IntDL, dorsolateral hump; IntDM, dorsomedial crest; IntIC, interstitial cell groups; IntP, posterior interposed nucleus; IV, inferior vestibular nucleus; Lat, lateral cerebellar nucleus; LC, locus coeruleus; LV, lateral vestibular nucleus; Med, medial cerebellar nucleus; MedDL, dorsolateral hump; MV, medial vestibular nucleus; MVm, magnocellular part of MV; MVp, parvocellular part of MV; scp, superior cerebellar peduncle; SV, superior vestibular nucleus; un, uncinate fascicle; vLat, ventral part of the Lat; Y, group Y

The caudomedial aspect of the Med is surrounded by white matter and lies against the base of the nodulus. The rostral half forms the roof of the 4th ventricle, while its ventrolateral aspect reaches towards the Lat and touches upon the superior vestibular nuclei (Fig. 3, levels 11 and 12 and levels 15 and 16 respectively). Perhaps the most conspicuous subnucleus of the rodent Med is its prominent dorsolateral protuberance (MedDL), first described by Goodman *et al*. [31] but not yet described in non-rodent species. The MedDL is formed by a group of neurons that, from the main body of the nucleus, reaches far dorsolaterally into the white matter and partly overlies the IntP (Fig. 3). Recent evidence suggests that the MedDL may be subdivided into a rostral and a caudal cluster, each with different connections [20]. From the medial part of the nucleus emerge the axons that form the uncinate fascicle, which subsequently crosses the midline within the cerebellum to reach the contralateral Med, the vestibular nuclei, and regions of the reticular formation (see “Efferent Connections of the Cerebellar Nuclei”).

#### Interposed Cerebellar Nuclei

As mentioned in “History and Notes on Nomenclature,” it is important that the two separate parts of the Int should be recognized as having quite different connections. IntP is located in the roof of the 4th ventricle, where its caudal aspect can be easily recognized. Rostrally, its borders with the other three nuclear constituents are more difficult to recognize **(**Fig. 3, panels 6 and 7). However, the IntP remains ventral to the IntA as its lateral aspect makes way for the ventromedial part of the Lat. Medially, the IntP gradually resolves into several isolated, or interstitial, cell groups (Fig. 3, panels 8 and 9), located within the white matter bundle that separates the Med from the IntA [32]. The efferent fibers of the IntP take up a medial position within the superior cerebellar peduncle (scp).

The IntA emerges caudomedially as a group of cells dorsal to the medial aspect of the IntP. This so-called dorsomedial crest [19] extends rostrally in a lateral direction, where it connects to a conspicuous bulge, termed the dorsolateral hump. Like the MedDL, this dorsolateral hump appears to be rather specific for rodents. Although this cell group is usually seen as part of the IntA, arguments can be made to include it in the Lat (cf. Figure 7). In general, the IntA forms a mediolaterally oriented ribbon of neurons lying on top of, and contributing efferent fibers to, the dorsal aspect of the scp. It should be stressed that, despite the fact that the IntA and IntP are often aggregated as “the” Int, their connections and transcriptomic makeup are quite different, suggesting they subserve rather different functions [33–35].

#### Lateral Cerebellar Nucleus

The Lat in rodents comprises a ventromedial part with small cells that can be distinguished from a dorsolateral part consisting of larger cells. These parts are likely to be the analog of the macrogyric and microgyric parts, respectively, of the human dentated Lat (see Fig. 2) [24]. Centrally, it contributes its fibers to and curves around the lateral aspect of the scp (Fig. 3, panels 12 and 13). Caudally, the Lat emerges as a laterally protruding cell mass flanking the IntP (Fig. 3, panel 6). Slightly more rostrally, it expands in a medial direction taking the place of the lateral IntP (panels 8–10). Rostral to the IntP, the ventromedial Lat almost reaches the lateral-most aspect of the Med (Fig. 3, panels 8–10). More rostrally, the lateral vestibular nucleus drives a clear wedge between the Med and the ventromedial Lat (Fig. 3, panels 10 and 11). This part of the Lat, furthermore, lies on top of the group Y, which, interspersed between the lateral vestibular nucleus and the inferior cerebellar peduncle, can be divided into a dorsal part with connections resembling a cerebellar nucleus and a ventral part that is generally considered a part of the vestibular nuclear complex [36, 37]. More laterally, the Lat forms the roof of the floccular peduncle. Within the peduncle, scattered cells can be found that also invade the floccular white matter. These cells, in primates, with a somewhat higher cell density, constitute the basal interstitial nucleus [38]. It is doubtful if these cells should be considered part of the Lat as they seem to function as a part of the cerebellar cortex [39].

### Connections of the Cerebellar Nuclei

Despite a wealth of literature on the subject, a comprehensive description of all the ins and outs of the CN cannot yet be given. This is due to the diversity of CN cell types (see “Cell Types of the Adult Cerebellar Nuclei”) in combination with the complex organization of both the terminal distribution of diverse groups of afferents (Table 2) as well as of the wide and complex distribution of CN efferents (see “Efferent Connections of the Cerebellar Nuclei”). Much of this complexity is expected to relate to the plethora of functions to which the CN likely contribute [40]. Hence, this section will only present a synopsis of CN connectivity.

**Table 2:** Overview of extracerebellar afferents to the CN. This overview is not intended to be exhaustive

CNS origin listed by projection type	Origin	Med	IntP	IntA	Lat	Cereb. cortex	Species	Remarks and references
Climbing fiber CN branches								
Medulla oblongata	Inferior olive	+	+++	++	+++	+++	Cat, rat	1, 2, 3
Mossy fiber CN branches								
Spinal cord	Cervical cord	++	++	++	0	+++	Rat	4
	Thoracic cord	++	++	++	0	++	Rat	5
	Column of Clarke	+	0	+	0	+++	Mouse	6
	Upper lumbar cord	++	++	++	+	+++	Rat	7
	Sacral and lower lumbar cord	++	+	+++	0	+++	Rat	4, 7
Medulla oblongata	External cuneate nucleus	0	0	0	0	++	Rat	8, 9
	Cuneate/gracile nucleus	0	0	0	0	++	Cat, rat	9, 10
	Spinal trigeminal nucleus	++	++	++	++	+++	Rat	11
	Lateral reticular nucleus	++	++	+++	+	+++	Rat	12, 13
	Paramedian reticular nucleus	++	++	++	++	+++	Rat	14
	Gigantocellular reticular nucleus	++	++	++	++	++	Rat	14
	Magnocellular reticular nucleus	++	++	++	++	++	Rat	14
	Vestibular ganglion	0	0	0	0	++	Cat, rabbit	15, 16
	Medial vestibular nucleus	++	0	0	0	++	Mouse	17
	Spinal vestibular nucleus	?	?	?	?	++	Rabbit	18
	Superior vestibular nucleus	?	?	?	?	++	Rabbit	18
Metencephalon	Basal pontine nucleus	0	+	0	++	+++	Rat	19
	Reticular tegmental pontine nucleus	+++	+++	+	+++	+++	Rat	19
Other								
Medulla oblongata	Caudal raphe interpositus	+	++	+	++	0	Rat	?, Not mono-aminergic; 20
Metencephalon	Pedunculopontine tegmental nucleus	++	++	++	++	++	Rat	Cholinergic; 21
	Dorsal tegmental nucleus	++	++	++	++	++	Cat	Serotonergic; 22
	Dorsal raphe nucleus	++	++	++	++	++	Cat	Serotonergic; 22
	Locus coeruleus	++	++	++	++	++	Cat	Noradrenergic; 23
Mesencephalon	Magnocellular red nucleus	0	+	++	+	0	Mouse/rat	Collaterals of rubrospinal neurons; 24, 25
Diencephalon	Tuberomammillary nucleus and hypo-thalamic regions	++	+	+	0	++	Rat	(mostly?) Histaminergic; 26,27

0 no, or very scant, projection; + sparse projection; +  + moderate projection; +  +  + dense projection; ? no information. References: 1: [41]; 2: [42]; 3: [43]; 4: [44]; 5: [45]; 6: [46]; 7: [47]; 8: [48]; 9: [49]; 10: [50]; 11: [51]; 12: [52]; 13: [53]; 14: [54]; 15: [55]; 16: [56]; 17: [57]; 18: [58]; 19: [59]; 20: [60];21: [61]; 22: [62]; 23: [63]; 24: [64]; 25: [65]; 26: [63]; 27: [66]

**Fig. 4:**
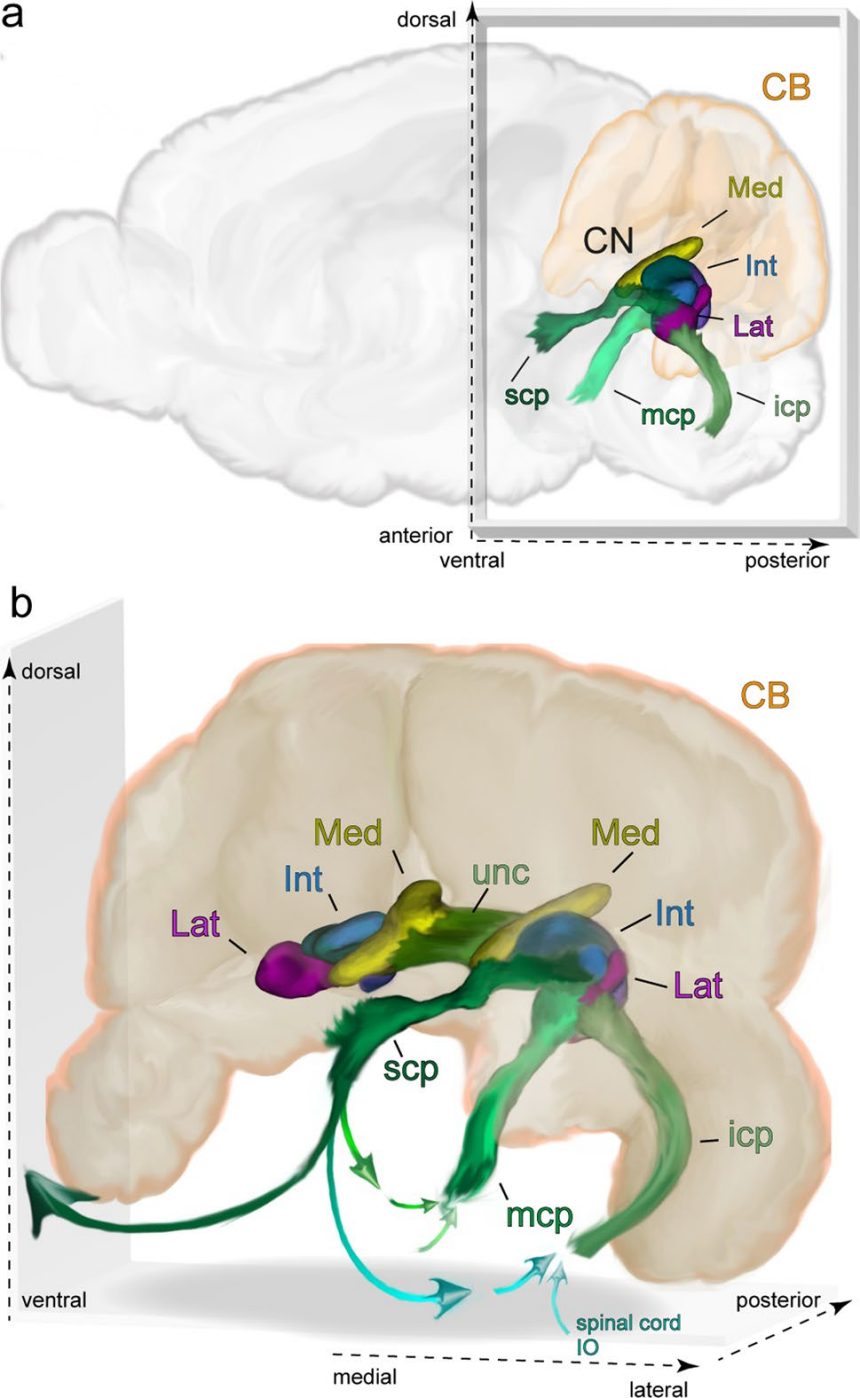
Overview of the CN of the mouse and the fiber bundles connecting them to the rest of the brain. **a** The location of CN and fiber bundles in a sagittal schematic of the mouse brain. **b** A depiction of the CN within the cerebellum. Arrows indicate the primary directions of axonal projections within the bundles. Dark green and light blue connections via the superior cerebellar peduncle indicate ascending and descending connections. The two arrows feeding into the icp and mcp indicate a combination of inputs arriving from ascending pathways (e.g., from the spinal cord or inferior olive) and descending ones (e.g. via the basal pontine nuclei). Note that, for clarity, the brainstem is not shown. Abbreviations: CB, cerebellum; Med, medial nucleus; Int, interposed nucleus; Lat, lateral nucleus; scp, superior cerebellar peduncle; mcp, middle cerebellar peduncle; icp, inferior cerebellar peduncle; unc, uncinate fibers; IO, inferior olive

Three massive fiber tracts connect the cerebellum with the rest of the brain, and these pathways also carry most of the signals to and from the CN (Fig. 4). The inferior cerebellar peduncle consists of afferents originating in the spinal cord and medulla, whereas the middle cerebellar peduncle carries fibers from the pontine nuclei to the cerebellum. Finally, the scp consists of the efferent fibers of the CN, particularly those from the IntP, IntA, and Lat. Some spinocerebellar systems also may reach the cerebellum by way of fibers overlying the scp [46, 67]. A fourth, somewhat smaller, bundle is formed by the uncinate fascicle, through which some of the efferent neurons in the medial half of the CN reach their extracerebellar targets.

#### Afferents of the Cerebellar Nuclei

**Fig. 5:**
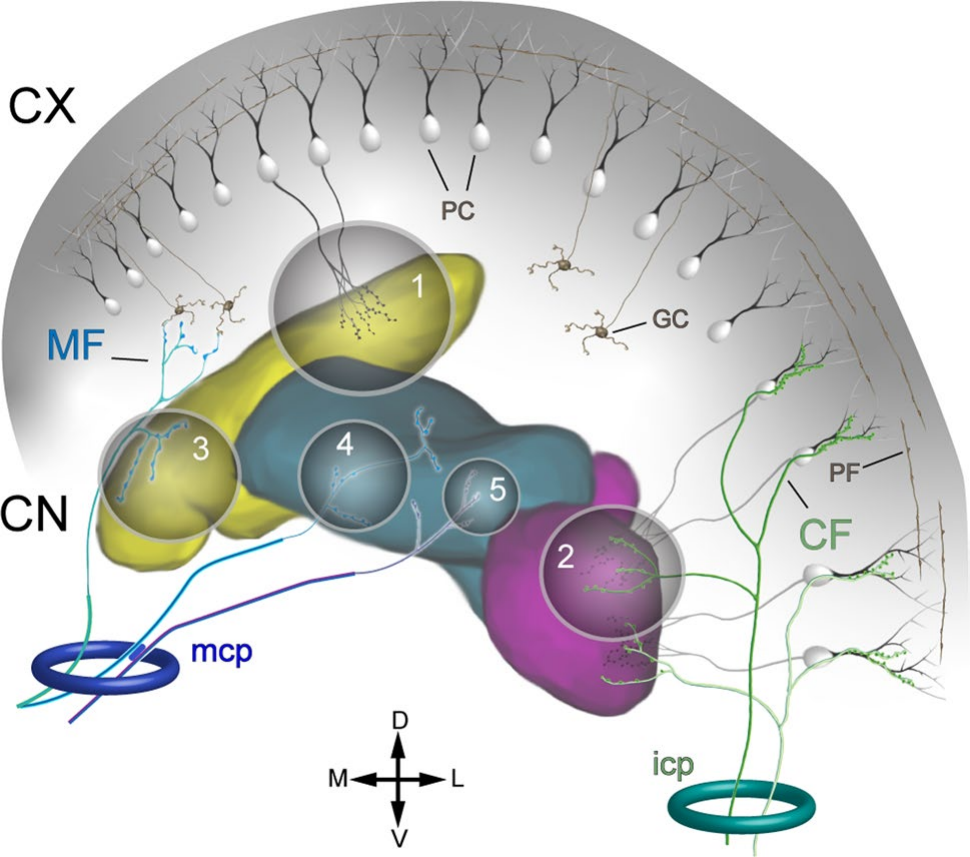
Schematic depiction of the 5 classes of CN afferent inputs, indicated by numerals: 1, GABAergic axons of the PCs converging on CN neurons; 2, glutamatergic axons of the IO neurons; 3, glutamatergic non-IO-originating axons that also branch as mossy fibers in the cerebellar cortex; 4, glutamatergic non-IO-originating axons that do not contribute to the cerebellar cortical mossy fibers; and 5, modulatory afferents. Abbreviations: MF, mossy fibers; PC, Purkinje cells; GC, granule cell; PF, parallel fiber; CF, climbing fiber; mcp, middle cerebellar peduncle; icp, inferior cerebellar peduncle. The arrows indicate approximate image directions: D, dorsal; V, ventral; L, lateral; M, medial. The Med, Int, and Lat are colored as in Fig. 2. For details regarding the distribution of the afferent axons among the nuclei, refer to Table 2

Afferents of the CN can be separated into two main types: (1) GABAergic input from the axons of PCs and (2) mostly excitatory input from extracerebellar sources. This second group can be further divided into (i) branches of CF originating from the inferior olive (IO); (ii) branches of afferents that terminate as mossy fibers (MF) in the granular layer of the cerebellar cortex; (iii) afferents from precerebellar neurons that do not terminate in the cerebellar cortex, and (iv) afferents from well-known modulatory systems (Table 2). Examples of CN afferents that escape this classification are the contralateral CN projections [68] and potentially the internuclear connections. Although local interneurons as well as local (recurrent) collaterals of projection neurons have been described [69], internuclear connections have not yet been reported as a prominent feature of internal CN organization. An overview of the extracerebellar sources of cerebellar afferents is provided in Table 2 and illustrated schematically in Fig. 5.

#### Corticonuclear Afferents

The CN are under inhibitory (i.e., GABAergic) influence of PCs originating from most of the cerebellar cortex. PCs from parts of the vermal and floccular cerebellar cortex also project to extracerebellar regions such as the vestibular and parabrachial nuclei [9, 10]. PC axons synapse on both excitatory and inhibitory neurons of the CN and vestibular nuclei [70].

The corticonuclear connectivity displays a clear-cut longitudinal patterning that was first recognized in the cat [71]. The axons of discrete, parasagittally organized stripes of PCs form specific white matter fascicles to the CN. As such, the longitudinal midline stripe of PCs that projects its axons to the Med is designated as the “A” module and characterized molecularly as the P1 ^+^ and P1 ^−^ stripes [summarized in 72]. Next to the A module, the PCs of the ‘B’ physiological module connect to the lateral vestibular nucleus; ‘C’ areas consist of several stripes of PCs that project to different regions of the Int and, finally, the PCs of the “D” modules connect to the Lat. “A” and “B” modules make up the vermis; “D” modules the hemispheres and in between these, the “C” modules constitute the paravermis or intermediate cerebellar cortex. This general pattern is also found in rodents and primates, and, especially for rodents, was later refined considerably once the molecular underpinnings were revealed (see “Cerebellar Modules”) [73–76]. In birds, striped PC patterns related to their target areas, clearly reminiscent of those found in mammals, have also been described [77–80]. Finally, it should be noted that PCs from parts of the vermal and floccular cerebellar cortex also project to extracerebellar regions such as the vestibular and parabrachial nuclei [9, 10].

#### Extracerebellar Nuclear Afferents: Primary Branches or Collaterals of Cerebellar Cortical Afferents?

The two main afferent systems of the cerebellar cortex, the MF and CF systems, also provide the bulk of the excitatory afferents of the CN. These nuclear fibers in the literature are usually referred to as collaterals of the MF and CF. However, when looking at these afferents from a nucleocentric point of view, it seems legitimate to ask the question if the CF and MF should be seen as the collateral branches of the primary cerebellar afferent that are directed to the CN, as we propose above in Fig. 1. Arguments supporting this view can be found in several observations. Indeed, as the nuclei constitute the output of the cerebellum, their excitatory afferents input will directly affect cerebellar output, leaving the MF-PC-CN pathway as a side loop (Fig. 1b). Also, axons from the trigeminal ganglia initially target the CN before they proceed to innervate the cerebellar anlage [81]. Later arriving olivocerebellar [82] and spinocerebellar axons [83] may distribute branches to the CN and cortical regions at the same prenatal time. As such, it has been speculated that spinal projection patterns to the nuclei resembling the adult organization predate adult terminal patterns in the cerebellar cortex. It therefore seems quite possible that the final fine-tuning of cortical CF organization may be based on functional connections made by their parent fibers in the CN.

An answer to the question if and how much the CN determines cerebellar cortical organization will only come once developmental and evolutionary research (see “Development of the Cerebellar Nuclei” and “Evolutionary Origins of the Cerebellar Nuclei”) determines the extent to which the patterning of cerebellar cortical afferents critically depends on the organization of the CN afferents (or vice versa). In addition, information is required on how, and to what degree, both the CN and cortical connections by the same afferent fiber might be adapted based on post-natal functional development [e.g., 84]. As this interesting set of questions is presently not resolved, we will avoid the use of the term “collaterals” in the remainder of this review (also see Fig. 1).

#### Olivonuclear Afferents

As stated above, CN afferents originating from the IO are considered to be branches of the olivocerebellar pathway that terminates in the cerebellar cortex as climbing fibers (CF) [43, 85]. These branches specifically target areas of the CN that receive the input of the PCs targeted by the same CFs. Thus, it can be stated that the organization of the olivonuclear afferent system adheres to the olivo-cortico-nuclear organization, culminating into a pattern of interconnected olivo-cortico-nuclear modules [76]. Although the olivary innervation seems to cover the entire contralateral CN complex, there are conspicuous differences with respect to the density of the terminal arborizations. For the rat, the ventromedial part of the Lat seems to contain the highest density of varicosities, potentially contributing to up to half of the available synapses [43, 86], whereas in other areas the density of olivary synapses has been estimated at 5–10%. Olivary afferents have been shown to terminate predominantly on dendrites of large projection neurons and small olivary projecting neurons [42, 87] (see “Cell Types of the Adult Cerebellar Nuclei” for a discussion of CN cell types).

#### CN Connections of Mossy Fibers

A major group of CN afferents originate as CN-directed branches of MFs, which, by themselves, constitute the most prominent input to the cerebellum. However, not all MF sources provide projections to the CN. Also, different parts of the CN may receive projections with a different density from a particular source of MFs. Table 2 shows a tentative and subjective (as concerns the numerical density) overview of MF innervation to the various parts of the CN complex. In general, it can be said that systems that deal with rather direct cutaneous or proprioceptive information (e.g., the column of Clarke, the dorsal column nuclei) provide no, or only scant, projections to the CN [46, 49, 88]. Conversely, MF systems originating from other parts of the spinal cord and the medulla (e.g., reticular and vestibular nuclei) are prominent sources of CN innervation (Table 2) [53, 57]. For the sake of simplicity and following past convention, though, we will here refer to the afferents as “MF.”

The MF afferents reach the cerebellum mostly by way of the inferior cerebellar peduncle, although some (e.g., a subpopulation of spinocerebellar fibers) take a route by way of the scp [46]. Additional CN-directed branches of MF afferents enter the cerebellum via the middle cerebellar peduncle and originate from the pontine nuclei, the main gateway of information from the cerebral cortex to the cerebellum. It should be noted that the CN contribution of the reticular tegmental nucleus heavily outweighs that of the basal pontine nuclei [59, 89, 90]. It, furthermore, is remarkable that the cortico-ponto-cerebellar projection ranks among the largest connection within the brain, but is incredibly difficult to chart and understand [91–93] and will not be further discussed here.

Many of the MF branches to the CN terminate bilaterally albeit with either contralateral (from pontine-originating MFs) or ipsilateral (e.g., from spinocerebellar MFs) preponderance, in contrast to the strictly contralateral localization of CN terminals of the IO axons. Furthermore, the MF arborizations are spatially less constricted in the CN than the IO axons in accordance with the more widespread distribution of MF rosettes over the cerebellar cortex as compared to the CF system [89, 94, 95]. CN terminals from MF are found on both small and large diameter dendrites of the large projection neurons [43, 96], but information is not available concerning their termination on other CN cell types (see “Cell Types of the Adult Cerebellar Nuclei”).

#### Other Nuclear Afferents

Special mention should be made of three afferent systems that do not seem to fit in any of the other groups. Glutamatergic rubrospinal projections have been demonstrated to terminate selectively in the IntA, without supplying MF or other fibers to the cerebellar cortex [64]. This suggests that a specific class of precerebellar premotor signals can influence cerebellar output without modulation via the cerebellar cortex [65]. Similarly, a region of the medullary reticular formation, referred to as the caudal raphe interpositus area, sends non-monoaminergic fibers to the CN without targeting the cerebellar cortex [60].

Finally, a direct projection from the primary somatosensory cortex to predominantly the ipsilateral CN, entering the cerebellum by way of the scp and demonstrating somatotopical features is transiently present in the neonatal cat [97]. Other such transient cerebral projections, however, have been described to reach the cerebellar cortex predominantly by way of the contralateral inferior cerebellar peduncle [98]. Although it has not been established if these CN and cortical projections are collaterals of the same axons, both projections seem to be temporary collaterals of persisting pyramidal tract axons [99]. In rodents, a sparse direct cerebral connection to CN and cerebellar cortex may be maintained into adulthood [100].

#### Monoaminergic and Cholinergic Nuclear Afferents

Here, only a brief overview of neuromodulatory afferents of the CN will be given. A dense serotonergic network of terminal fibers arises from the dorsal raphe nucleus, dorsal tegmental nucleus, and serotonergic cells within and around the locus coeruleus as well as from several other pontomedullary nuclei [62, 101]. Serotonin has been shown to have down-regulating effects on both GABAergic and glutamatergic synapses within juvenile rat CN [102–104]. Moreover, serotonergic innervation has been suggested to be involved in the development of normal adult cerebellar function [105].

A noradrenergic projection to the CN originates at least partly from the locus coeruleus [106]. Although the projection density seems to be rather uniform across all CN, differences in the density of adrenergic alpha and beta receptors among the nuclei are thought to underlie opposing effects of noradrenergic modulation [107, 108]. A system of cholinergic fibers with a variable density throughout the CN originates from the pedunculopontine and laterodorsal tegmental nuclei, as well as from the vestibular nuclei [61, 109]. Finally, although dopamine receptors have been shown to be broadly expressed in the CN in various cell classes [110], the source of dopaminergic innervation is not yet established. Curiously, the ventral tegmental area provides a dopaminergic projection to the cerebellar cortex but its projection to the CN has been described as glutamatergic and not dopaminergic [111].

Apart from the monoaminergic innervation, the CN also receive fibers containing neuropeptides such as corticotropin-releasing factor, enkephalin, cholecystokinin, and orexins, which at least partly co-localize as members of the MF and CF pathways [112–117]. In general, very little is known about the specific functional impact of these neuromodulatory systems on CN function [118].

#### Cerebellar Modules

**Fig. 6:**
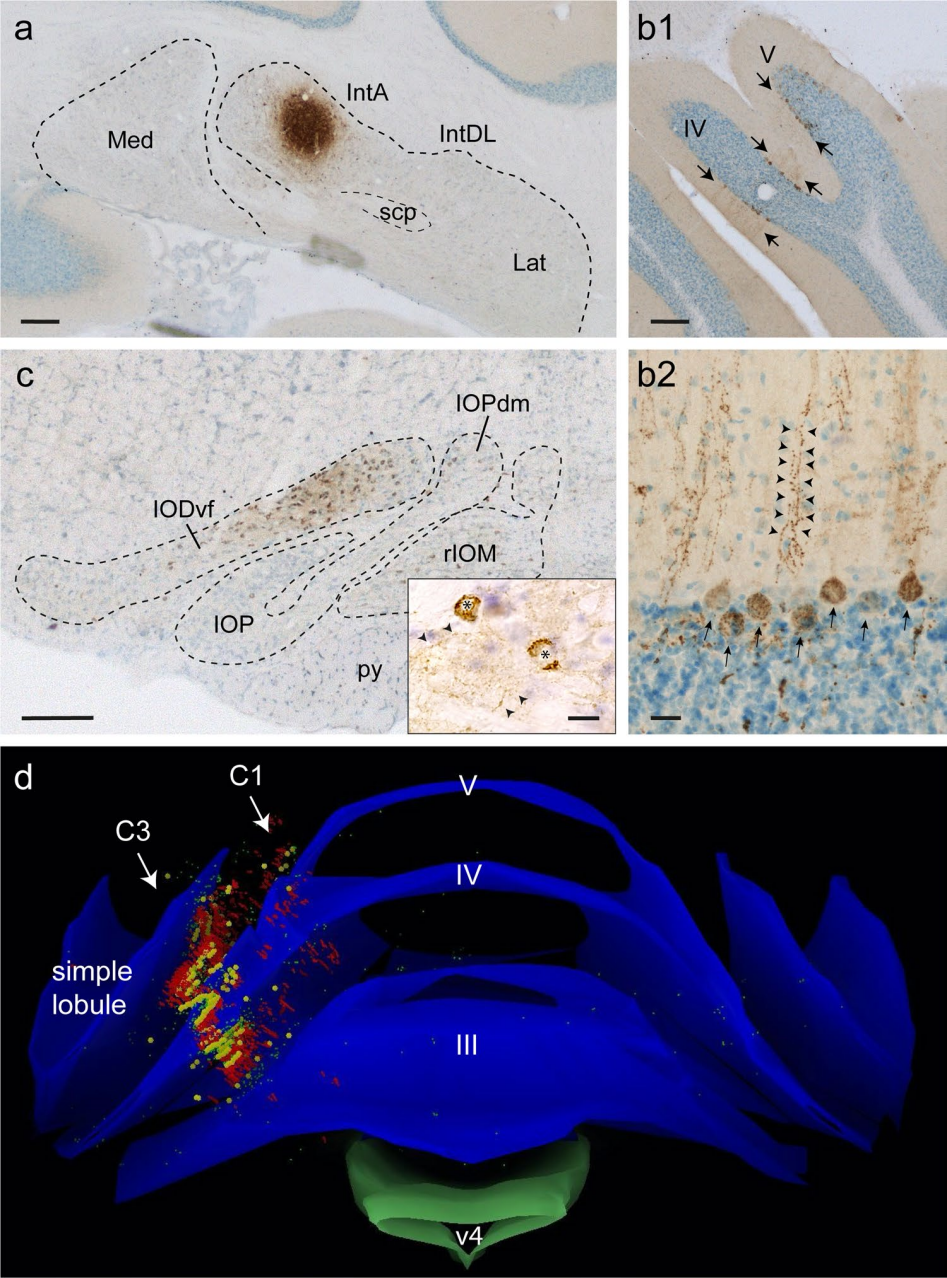
Illustration of modular connections in the rat cerebellum. **a** Iontophoretically applied injection of cholera toxin centered on the IntA, without involvement of surrounding nuclei. **b1** A stripe-like band of retrogradely labeled PC’s in lobules IV and V of the anterior lobe. **b2** Detail of cortical labeling showing retrogradely labeled PC somata aligned with CF terminals running like railroad tracks perpendicular to the surface in the molecular layer. **c** Retrogradely labeled olivary cells are only observed in the ventral fold of the dorsal accessory olive. Inset shows detail with labeled olivary neurons (asterisks) and dense labeling of fine terminal arborizations of nucleo-olivary afferents in the neuropil (between arrowheads). **d** 3D reconstruction (Neurolucida™) showing the white matter (blue) of the anterior part of the cerebellum (seen from the anterior) with the location of labeled PCs (yellow) and labeled CF (red). Note the near-perfect correspondence of both types of labeling indicating the modularity of the olivo-cortico-nuclear connections. Scale bar equals 250 μm in **a**, **b1**, **c** (10 μm in inset), 25 μm in **b2**. Abbreviations: III, IV, V, cerebellar lobules III, IV, V; C1, C3, PC stripes projecting to IntA; IntA, anterior interposed nucleus; IntDL, dorsolateral hump; IODvf, ventral fold of dorsal accessory olive; IOPdm, dorsomedial group of the principal olive; IOP, principal olive; Lat, lateral cerebellar nucleus; Med, medial cerebellar nucleus; rIOM, medial accessory olive, rostral part; scp, superior cerebellar peduncle; v4; fourth ventricle [Modified from 30]

Apart from its cytological appearance (see “Morphology of the Murine CN Complex”), the rodent CN can be further subdivided into smaller units based on their connections and biochemical identity of the PCs. By forming a robust and finely detailed reference frame, the striped pattern of the zebrin II/Aldolase C of the PCs [73,119–125] greatly helped in determining the highly detailed, fine-grain organization of olivo-cortico-nuclear connections with matching olivonuclear and nucleo-olivary projections [75, 76, 121,126–129]. An example illustrating the matching organization of olivo-cortico-nuclear interconnectivity is shown in Fig. 6 by a small injection with the neuroanatomical tracer ß-subunit of cholera toxin into a part of the IntA. As the tracer is transported both retrogradely (labeling PCs and inferior olivary neurons) and anterogradely (labeling nucleo-olivary terminals, but also olivocortical CF), it can be appreciated that the CF distribution nicely matches that of the retrogradely labeled PCs.

**Fig. 7:**
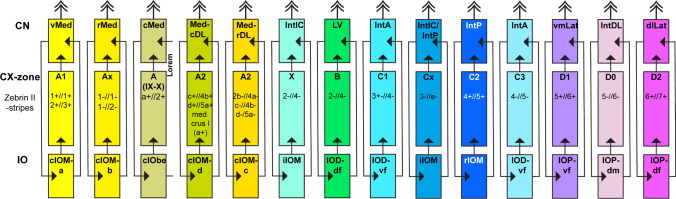
Schematic representation of the olivo-cortico-nuclear interconnectivity. Note that each module, apart from their interconnected parts, also connects to the rest of the brain by their output (double arrowheads). Also note that the cortical part of each module is formed by one or several stripes of either zebrin II + or zebrin II- Purkinje cells. The mediolateral order of the represented modules, indicated from left to right in the diagram, is based on the mediolateral position of the CN and is not related to the mediolateral position of the cortical components. For example, the cortical A2 modules are located lateral to the cortical B-module [see 138]. IntDL receives input from zebrin II-negative PCs of cortical module D0, which is interspersed between zebrin II ^+^ stripes D1 and D2 and receives its olivary input from a part of the principal olive (IOPdm). Different shades of yellow, blue, and purple refer to Med, Int, and Lat modules, respectively. The green module is related to the lateral vestibular nucleus (LV). Note that modules of the vestibulocerebellum, i.e., with cortical input form nodulus and flocculus, are not indicated in this scheme. Abbreviations. Inferior olive (IO) per module from left to right: cIOMa, group a of caudal medial accessory olive (cIOM); cIOMb, group b of cIOM; cIOMbe, group beta of cIOM; cIOMd, group d of cIOM; cIOMc, group c of cIOM; IODdf, dorsal fold of dorsal inferior olive (IOD); IODvf, ventral fold of IOD; iIOM, intermediate part of medial accessory olive (IOM); rIOM, rostral part of IOM; IODvf, ventral fold of IOD; IOPvf, ventral fold of principal olive (IOP); IOPdm, dorsomedial group of IOP; IOPdf, dorsal fold of IOP. CX-zone: sagittally oriented zones of Purkinje cells in the cerebellar cortex indicated by capital letters A to D followed by either a number or a lowercase ‘x’ and related to zebrin-positive or zebrin-negative stripes. Cerebellar nuclei (CN) from left to right: vMed, ventral part of medial cerebellar nucleus (Med); rMed, rostral part of Med; cMed, caudal part of Med; MedcDL, caudal part of dorsolateral protuberance of Med; MedrDL, rostral part of dorsolateral protuberance of Med; IntIC, interstitial cell groups; LV, lateral vestibular nucleus; IntA, anterior interposed nucleus; IntIC, interstitial cell groups; IntP, posterior interposed nucleus; IntA, anterior interposed nucleus; vLat, ventral part of lateral cerebellar nucleus (lat); IntDL, dorsolateral hump; dLat, dorsal part of the Lat. Based on [20, 72, 139]

In rodents, based on expression domains and matching connectivity patterns, at least 14 modules have been recognized [20, 72, 129] (see Fig. 7). Note that in rodents, likely related to the remarkable rodent proliferation of part of the Med in the dorsolateral direction (MedDL), a cortical part of the A module (A2) is located lateral to the B-module in the paravermis, which contrasts the situation in carnivores and primates, where no Med projecting PCs are found lateral to the B-zone [20, 126]. Some CN regions may receive PC afferents from several cerebellar cortical stripes (e.g., C1 and C3 module to IntA) [131]. Additional cortical stripes in the ventral uvula/nodulus and flocculus have been described as specific sources in their projections to other parts of the vestibular nuclei or selective regions of the CN [9]. Furthermore, for some modules, physiological data suggests that further subdivisions into longitudinally oriented micromodules each with its own peripheral receptive field are possible [e.g., 132–134]. The same is true of parasagittal stripes defined by PC expression markers—stripes that appear unitary in one expression map show further subdivisions when examined with other markers [e.g., 135, 136]. However, it is not known to what extent the modular circuitry (i.e., relating to all connections within the olivo-cortico-nuclear loops) remains parallel and non-overlapping at the microzonal level. Available anatomical evidence suggests that for some modules finer subdivisions indeed may exist [137].

This question has become more pressing, as an added level of complexity exists beyond the rather straightforward parasagittal organization of the corticonuclear projection that forms the basis of the modular organization. As detailed studies on the connectivity, function and gene expression profiles of the cerebellar cortex indicate, transverse cortical boundaries exist as well [72, 119, 140]. At least four such transverse zones have evolved in mammals—the anterior zone (comprising mainly lobules I–VI), the central zone (lobules VI–VII) [141], the posterior zone (lobules VII–VIII) and the nodular zone (lobules IX–X) [142]. The cortical parasagittal stripes and transverse zones all can be further subdivided into numerous small regions based on their patchy MF afferent fields corresponding to molecular heterogeneities of MF synapses in the granular layer [143, 144]. Indeed, the number of discrete cortical compartments has been estimated to reach several thousands [72, 123]., However, despite the division of the cerebellar cortex into numerous parasagittal stripes and transverse zones, the cortical connections to the CN seem much more simple as the Zebrin II-positive Purkinje cells target the caudoventral aspects of the CN, whereas the Zebrin II-negative cells project to its rostrodorsal parts, thereby dividing the CN in a basically Zebrin II-rich and a Zebrin II-poor area [75], suggesting that a high-grained parcellation of the CN resembling that of the cortex does not exist. It is, therefore, unlikely that the same level of compartmentalization exists in the CN, which therefore may receive input from many, up to more than one hundred, of these discrete cortical compartments. Unfortunately, at present virtually no information is available that indicates the computational role of these CN entities [72].

It should also be borne in mind that the corticonuclear pathways are not independent at the cerebellar cortical level—there is substantial crosstalk via the parallel fiber system. In this respect, the role of the distributed input of precerebellar (MF) information to the nuclei as well as to the cerebellar cortex is far from being resolved. Nevertheless, despite these questions concerning the fine architecture of these olivo-cortico-nuclear circuits, their basic modular organization has been proposed to form functional cerebellar entities that, by way of their module-specific output, can modify ongoing or future processing of specific functions [72].

#### Efferent Connections of the Cerebellar Nuclei

**Fig. 8:**
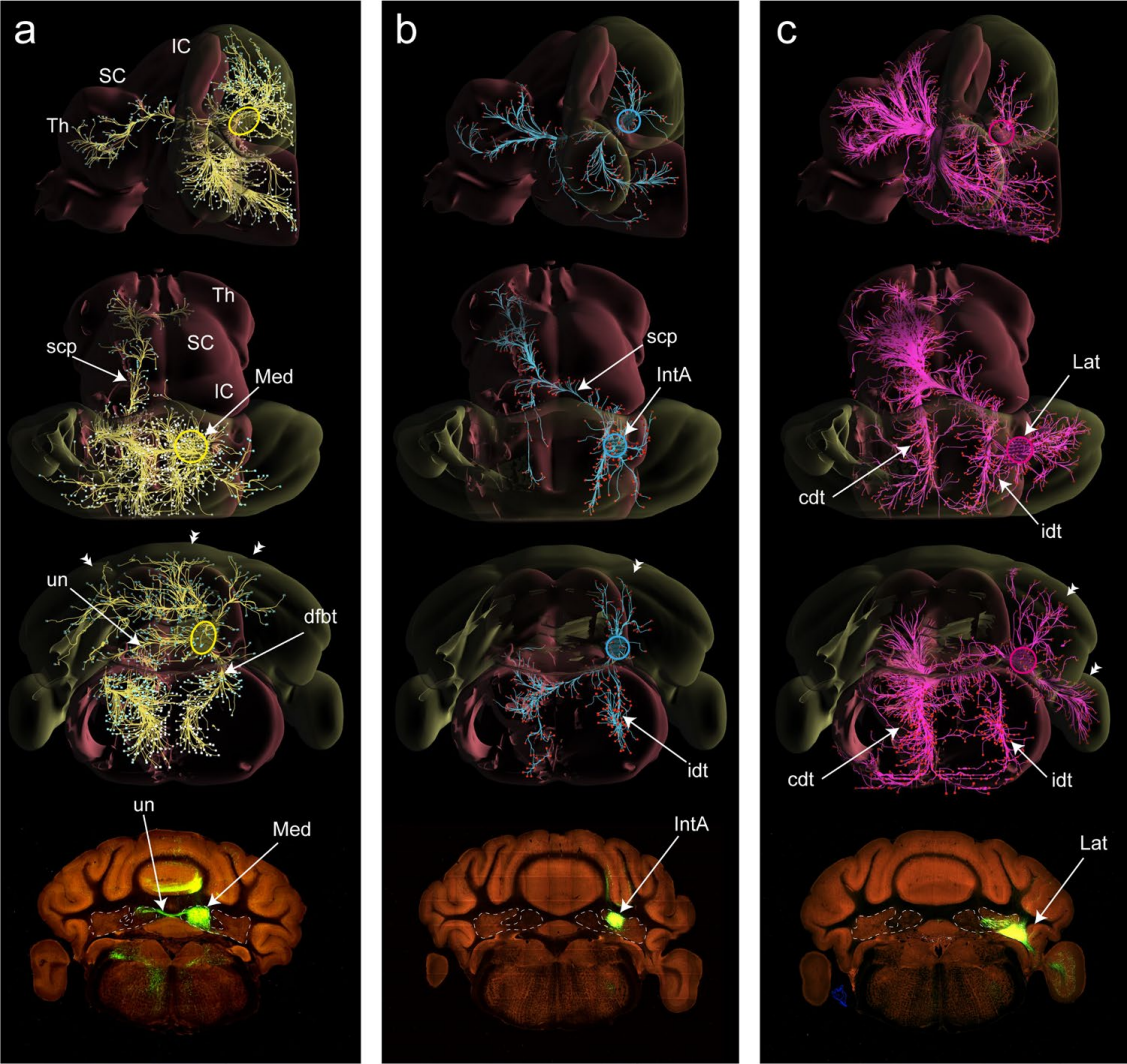
3D representations of CN projections to the brainstem and thalamus in the mouse visualized using recombinant anterogradely transported adeno-associated virus (rAAV) as a tracer injected in different parts of the CN. Panels from top to bottom depict a lateral, dorsal, and caudal transparent 3D view with a final section showing the injection site. **a** Injection centered on the Med, depicting prominent bilateral terminal labeling in the vestibular nuclei and medial reticular formation. Note the conspicuous intracerebellar course of the uncinate fascicle. **b** Injection centered on the IntA. Apart from the course of labeled fibers to the midbrain and thalamus by way of the scp, note the prominent course of ipsilaterally descending fibers, which seems to be a special feature of rodent connectivity. **c** Injection centered on the Lat. Here, aspects of an ipsilateral descending tract together with a contralateral descending tract terminating in the medullary reticular formation can be appreciated. See text for further explanation. Yellow, cyan, and magenta circles indicate the approximate sites of the Med, IntA and Lat injections respectively, in the 3D representations. Double arrowheads in the third row point to the nucleocortical projections seen in all cases. Abbreviations: cdt, contralateral descending tract; dfbt, direct fastigiobulbar tract; IC, inferior colliculus; idt, ipsilateral descending tract; IntA, anterior interposed nucleus; Lat, lateral cerebellar nucleus; Med, medial cerebellar nucleus; SC, superior colliculus; scp, superior cerebellar peduncle; Th, thalamus; un, uncinate fascicle; based on material from [145], experiment numbers 268389532, 120, 493, 315, 127, 650, 431)

The efferent projections of the CN have been studied with a variety of techniques and demonstrate a complex organization. Although classically the CN were seen as projecting to the motor regions of the thalamus, some premotor nuclei in the brainstem, and the IO, it now has become clear that the CN influence a multitude of very diverse targets in the diencephalon, brainstem, and spinal cord. These targets are reached by different pathways. As an example, Fig. 8 shows a 3D composite of labeled fibers and terminal branches resulting from small anterogradely transported viral tracer injections into the Med, Int, and Lat parts of the mouse CN, which can be constructed from the Allen Brain Atlas website [145]. From these reconstructions, it can be appreciated that the efferents from the three injection sites not only distribute to many regions of the brain, but do so by taking different routes. Additional detailed examples of many injections for primate, rat, and mouse can be found in the literature [20, 35, 146, 147].

The axons of most projection neurons of both IntP and Lat CN, supplemented by some fibers from the Med, leave the cerebellum by way of the ipsilateral scp. Upon entering the pontine tegmentum, a sizable number of axons, mostly originating in the dorsolateral hump and surrounding areas, exit the scp laterally to form an ipsilaterally descending tract (Fig. 8b, c) that terminates in the pontine and medullary parvocellular reticular formation and within the spinal trigeminal nucleus [147, 148]. Some fibers have been described to descend as far as the ipsilateral lumbar cord [149].

The main part of the scp decussates in the mesencephalon, where it divides into a major contralateral ascending tract, still referred to as scp, and a smaller contralateral descending tract (Fig. 8b, c). The contralateral descending tract (Fig. 8c) carries fibers to the pontine nuclei, pontine and medullary reticular formation, and IO. The Lat in particular sends a major projection to the contralateral medulla by way of this tract. The projections to the IO originate from small GABAergic neurons [150, 151] that are distributed throughout the CN where they are intermingled with other CN neurons [35, 151–154]. The axons of these nucleo-olivary neurons ascend in a loose bundle just ventral to the medial aspect of the scp towards its decussation in the midbrain before turning caudally to reach the IO from a position just dorsal to the lateral part of the pyramidal tract [153, 156, 157]. Once entering the target olivary nucleus, the contralateral nucleo-olivary axons branch explosively in a conical shape, forming dense volume-filling meshes of synaptic terminals [158, 159]). Some nucleo-olivary fibers recross the midline at the level of the IO to form more diffuse terminal fields ipsilateral to the injection site [157, 160]. The contralateral ascending tract of the scp, carrying the main bulk of excitatory fibers from the CN, sends its projections to the superior colliculus, many regions in the midbrain tegmentum (e.g., the red nucleus), periaqueductal grey, pretectum and many thalamic and several hypothalamic nuclei [35, 40, 147].

Many efferents from the Med, as well as from a small population of spinal cord projecting neurons in the Int [149], take an alternative route to the brainstem. These fibers cross within the cerebellar white matter and enter the contralateral uncinate fascicle that arches dorsal to the contralateral scp in order to reach the contralateral vestibular nuclei and medial reticular formation and, in some cases, the cervical spinal cord. Viral tracing techniques indicate that these direct cerebellospinal projections originate from the Med and IntP [149]. Before crossing over the scp, a sizeable portion of the uncinate fascicle enters the most medial aspect of the contralateral scp as its crossed ascending limb [36, 161]. This bundle does not recross in the decussation of the scp, but remains contralateral to its origin and sends terminal branches to regions of the mesencephalic reticular formation, periaqueductal grey, superior colliculus, and thalamus (Fig. 8a). Some, mostly GABAergic, Med fibers cross in the roof of the 4th ventricle and have terminals in the contralateral Med [68]. Fibers of the Med that do not follow the uncinate fascicle or the scp, pass medial to the ipsilateral scp to reach the ipsilateral vestibular nuclear complex and adjacent reticular formation by way of the direct fastigiobulbar tract [162] (Fig. 8a). These ipsilaterally projecting neurons are thought to be mostly glycinergic [163].

As the CN part of the modules are also at the origin of extracerebellar projections (Fig. 7), it would make sense to study the projections from the various modules. Recently, for the Med, at least five excitatory neuronal groups were recognized, which each seemed to participate in a striped arrangement of olivo-cortico-nuclear connections. Monosynaptic and transsynaptic tracer studies suggest that each of these Med groups projects to targets which subserve different functions. Hence, the ventrolateral Med (Fig. 7) projects to areas participating in postural control and coupling of locomotion and respiration, and general autonomic control; the rostral Med targets a number of posturomotor regions; the rostral MedDL seems to be related to oromotor control, whereas the caudal MedDL would subserve salience and orienting functions. Finally, the caudal Med would be at the origin of projections controlling vigilance [20].

Unfortunately, similar detailed studies of the Int and Lat are not available, although several studies linking anatomy to function of CN parts have become available [149, 164, 165]. Yet, as CN targets are found in a long list of regions from the spinal cord to diencephalon [35, 147], it would be expected that many projection neurons terminate in multiple areas. Indeed, extensive axonal branching has been established to many regions [35, 149,166–168]. This suggests that functionally related networks are linked by branches from selective groups of CN neurons [20, 35, 149]. Indeed, transneuronal studies suggest that individual muscles may be under the influence of several regions of the cerebellar cortex [169]. Similarly, functional regions of the cerebral cortex have been shown to be under the influence of multiple cerebellar modules [170]. It is clear that a comprehensive description of single-cell terminal field patterns of the different modular or micromodular groups of CN neurons would greatly help us understand the functional organization of the CN. Unfortunately, such a description is still lacking.

In describing the targets and terminal fields of the projection neurons of the CN, it should be recognized that a sizeable projection originating from excitatory projection cells is directed to the cerebellar cortex [171–173]. These nucleocortical projections terminate in the granular layer with a MF rosette-like morphology and have been suggested to produce internal amplification during motor learning [174]. An additional nucleocortical projection arising from Type 2 glycinergic neurons (as defined in Table 3) in the CN and mostly terminating with varicose terminals within the granular layer selectively inhibits a subpopulation of Golgi interneurons [175].

**Table 3:**
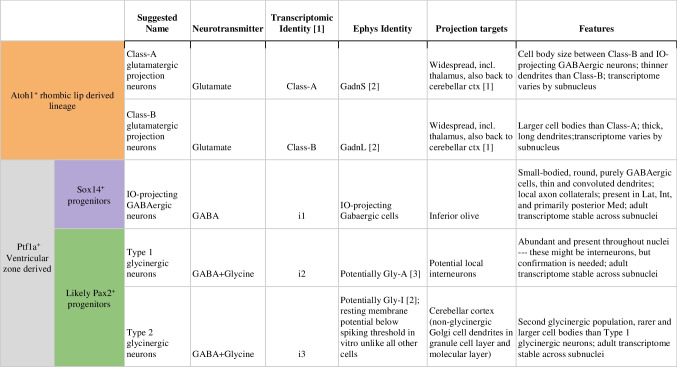
Cell type composition of the CN. Integration of transcriptomic, electrophysiological, and morphological data results in a set of five cell types that are present in each CN, and that are conserved across amniotes

Note that a small group of nucleus-specific glycinergic cells present in the lateral part of the Med in mice is not part of this Table, as, on the surface, it violates this rule. Closer inspection, however, suggests that it is likely a Class-B type neuron that switched neurotransmitter from glutamate to glycine, thus fitting into the scheme. References: 1:[35], 2:[176]; 3:[69].

It is well known that the cerebellum and cerebrum are reciprocally connected. Major pathways course from the CN to the thalamus and onward to the cerebral cortex and from there to pontine nuclei back to the cerebellum. Within these circuits, the cerebellar modular organization seems to be respected as closed loops between specific parts of the cerebral cortex and stripe-like regions of the cerebellar cortex [177]. However, partly open loops due to diverging and converging projections within the nucleocerebral as well as within the cerebrocerebellar routes also may exist suggesting interactions between (micro-)modules at extracerebellar locations [92, 170]. Moreover, several other subcortical excitatory recurrent circuits are effective, such as a reverberating nucleo-ponto-nuclear circuit, in which CN efferents activate neurons in the basal and/or reticular pontine nuclei that provide excitatory input to the CN, thereby maintaining excitation within the circuit [178]. A similar reverberating circuit is found in the projections of the IntA to the red nucleus, which in turn sends recurrent rubrospinal collaterals back to the IntA [65]. Finally, nucleo-midbrain-olivocerebellar circuits can be recognized that connect CN efferents from Lat and IntP with the primate parvocellular red nuclei and the rodent mesodiencephalic areas, which form an important input to parts of the IO [179]. The functional roles of these circuits are far from being established (also see section “Downstream Actions of Cerebellar Efferents”).

## Cell Types of the Adult Cerebellar Nuclei

### Historical Perspectives

In striking contrast to the well-established identification of neuronal types forming the cerebellar cortical circuitry, the classification of neuron types in the CN remains incomplete at best. Quite recently, technological advances in genetic targeting and neurite tracing have brought important novel insights into neuronal diversity (see below) and their functional significance (see “Physiology of the Cerebellar Nuclei”). However, the discourse on CN neurons in the current literature, as far as it is relevant for validating or developing theories of cerebellar function, is largely based upon only two classes of CN neurons assumed to be present in each part of the CN—the large, glutamatergic neurons that project to diverse regions outside of the cerebellum and the small, GABAergic neurons that project to the IO—providing a means for the cerebellum to modulate its key timing signal. While this binary classification has long been viewed as overly simplistic, methodological difficulties in identifying cell types in living animals have limited the scope of CN functional investigations. Here, we provide an overview of the most current view on the classification of CN cell types, in the hope that it will inspire extending the focus of future experiments beyond the broad classes of “glutamatergic” or “GABAergic” CN projection neurons and result in a more contemporary circuit analysis approach applied to its research.

Historically, CN neurons have been classified into two or three classes based on their soma sizes [19, 31, 180, 181]. These early studies were mostly focused on the Lat and offered little functional insight besides speculations on their projection targets and observations of differential distribution within CN regions, classically described as the “magnocellular” and “parvocellular” parts. Chan-Palay was the first to include detailed quantification of somatodendritic morphology and orientation in the definition of 6 classes of neurons in the rat and monkey Lat (4 classes of “large” and 2 classes of “small” neurons) [146].

The diversity of neuronal morphological classes and its implications for the physiology and function of the CN was largely unexplored in the initial decades of in vitro electrophysiological experimentation. Notably, in the first reports of electrical responsiveness of CN neurons [182–184], no evidence of differences among CN cell types was found and their ionic properties, characterized by spontaneous generation of action potentials, spike afterpotentials, plateau potentials, and rebound spiking, were considered to be identical. In retrospect this is not surprising as the CN in vitro slice preparation later turned out to be one of the most challenging ones in CNS research, limiting the results to certain cell types in juvenile animals [185]. It also might be surprising to younger readers that in the past it was not obvious that any neurons in the CNS would have different electrical properties, and even less so what degree of similarity and variability might be expected between different types of neurons. The first insights into electrophysiological variability among cells, manifested by differences in bursting behavior, were described by Aizenmann and Linden [186]. Analogous to the electrophysiological signatures of neurons in the vestibular nuclei to which the CN are often considered closely related [187–189], the CN neurons were classified into two electrophysiological groups: fast-spiking, large neurons that were assumed to be the principal (projection) neurons of the CN, and smaller, slow-spiking neurons, thought to represent interneurons.

The advances in genetic targeting of living cells with fluorescent indicators in the first years of the 2000s drove a revolution in combining electrophysiological, morphological, and molecular fingerprinting of neurons. Thus, electrophysiological differences between CN neurons, in addition to morphometric features, could now be relatively unambiguously delineated by protein expression patterns in living slices. A series of studies of the CN starting from 2007 took advantage of reporter mouse lines to identify neurons based on the expression of markers associated with either GABAergic or glycinergic neurotransmitter phenotypes [GAD67 and GlyT2, respectively, 69, 163, 190, 191]. The resulting, at the time somewhat surprising revelation of GABAergic neurons expressing slower spike frequencies than putative glutamatergic neurons, was subsequently complemented by using increasingly specific genetic tools, such as viral transfection in combination with cre-lox expression systems, to differentiate axonal target regions [173, 175].

### Classification of Cerebellar Nuclear Cells

#### Neuron Classes

Recent comprehensive transcriptomic investigations in adult mice [20, 35] identified 14 nucleus-specific excitatory cell types within the CN, one nucleus-specific glycinergic cell type, and ~ 3 nucleus-invariant inhibitory and/or glycinergic cell types. Closer inspection of the diversity of excitatory cell types allows the grouping of these cell types into two cell type classes. Both classes are represented in each CN subdivision [“subnucleus” as defined in 35] and, at this level of classification, are shared across all nuclei. Each member of each class, however, also expresses subdivision-specific transcriptional signatures on top of their class-specific gene expression profiles, making them recognizable as distinct cell types [35]—thus yielding a total of 14 excitatory cell types across the CN. Interestingly, the nucleus-specific glycinergic cell type, which corresponds to large-bodied, glycinergic neurons that occur only in the rostrolateral part of the Med [163], appears transcriptomically, developmentally [35], and electrophysiologically [163] similar to one class of the glutamatergic projection neurons. For simplicity, we will therefore consider it effectively a glutamatergic neuron that has switched its neurotransmitter. The functional and evolutionary implications of this intriguing case need further investigations, however.

**Fig. 9:**
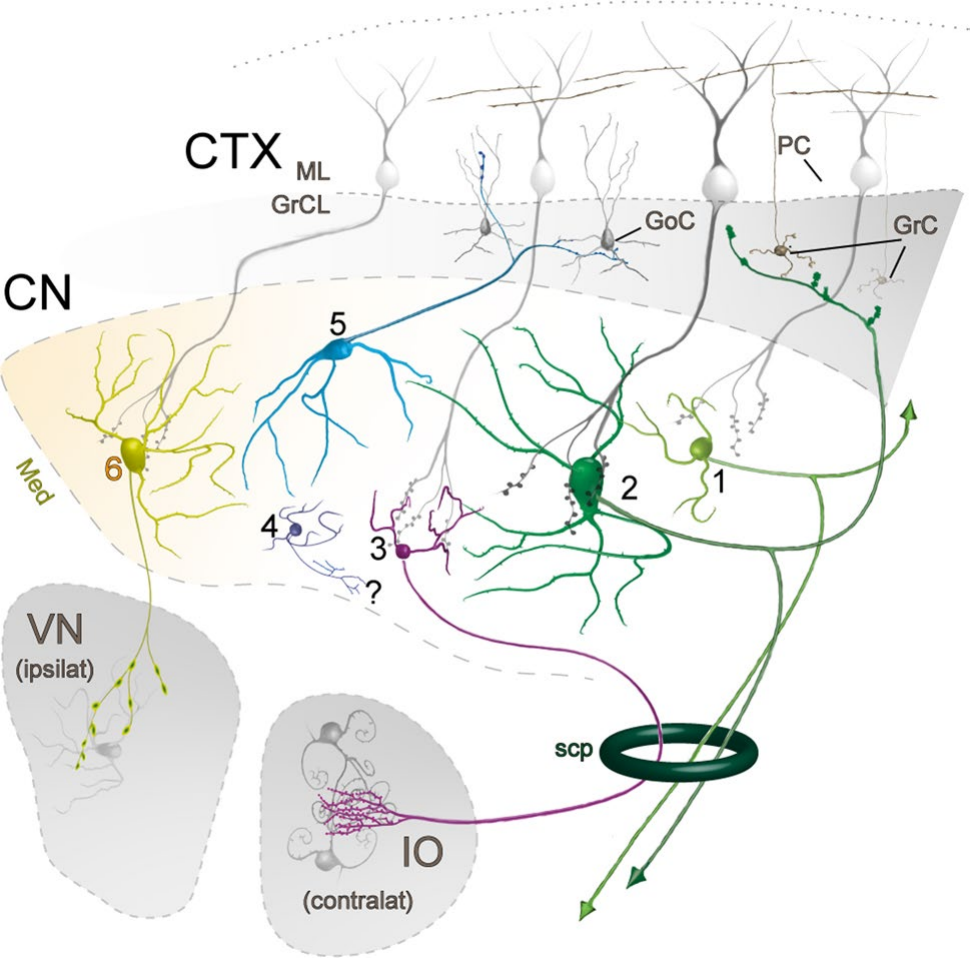
Schematic representation of the CN cell types and their connectivity. Numbers refer to descriptions in Table 3. Abbreviations: ML, molecular layer; GCL, granular layer; GoC, Golgi cell; PC, Purkinje cell; GC, granule cell; Med, medial CN (hosting the “exceptional” glycinergic projection neuron type labeled 6); VN, vestibular nucleus; ipsilat, ipsilateral; IO, inferior olive; contralat, contralateral; scp, superior cerebellar peduncle. The question mark indicates unknown targets of local interneuron axons

By weaving together the separate lines of evidence from neurotransmitter expression, morphology, spatial location, development, and limited patch-seq data, we here propose a canonical set of five neuron types that are present in every CN (Table 3 and Fig. 9) and are conserved across the amniotes [35]:

- Class-A glutamatergic projection neurons
- Class-B glutamatergic projection neurons
- IO-projecting GABAergic neurons
- Type 1, likely local, GABA and glycinergic neurons
- Type 2 cerebellar cortex-projecting GABA and glycinergic neurons

How the diversity of transcriptomic cell types across the CN corresponds to their diversity in projection targets and intrinsic properties is still largely unexplored, even though the functional profiles of CN neurons—such as differences in retrograde signaling and plasticity—likely lead to significant differences in the information that specific target regions would be receiving regarding cerebellar computation. In all, we expect that additional subdivisions in the hierarchical organization of CN cell types will be revealed by future integration of single-cell connectivity, electrophysiological fingerprinting, spatial location, developmental history, and gene expression data.

#### Glia in the Cerebellar Nuclei

**Fig. 10:**
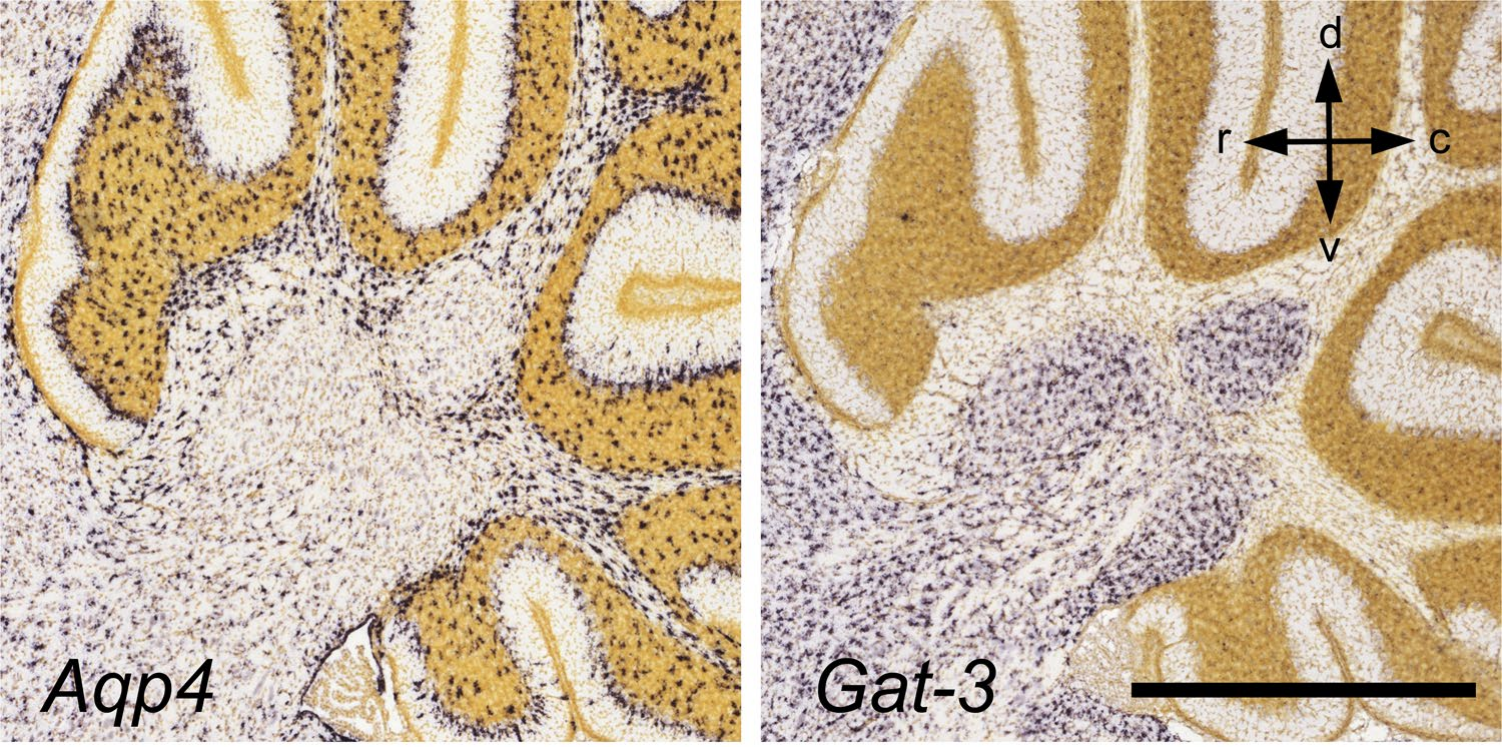
In situ hybridization demonstrating expression patterns of astroglial markers, *Aqp4* and *Gat-3*. Sagittal sections through the lateral vermis. CN are either negatively outlined by staining for *Aqp4*, or positively by staining for *Gat-3*. In contrast, astroglial cells in the white matter and the granule cell layer, and also Bergman glial cells, strongly express the mRNA for *Aqp4*, but not that for *Gat-3*. Arrows give orientation (r, rostral, c, caudal, d, dorsal, v, ventral. Scale bar = 1 mm. [145]

We are not aware of studies focusing on the glial cell biology of the CN. Chan-Palay [146] noted that astroglial cells outnumber neurons in the Int by a factor of ~ 8 in rats, and by a factor of ~ 12 in monkeys (*Macaca mulatta*). Even if these numbers may need some downward correction, as has been necessary for cerebellar cortical estimates over the past few years [see 192], the CN stand out as having a high glia-to-neuron ratio. Chan-Palay also pointed to morphological differences between astrocytes in the cerebellar cortex, white matter, and CN [146]. Expression of the classical astroglial marker, GFAP does not suggest any obvious differences in pattern and intensity between the CN and the granular layer [193, see also 194]. However, more recent immunocytochemical and gene-expression data indicate functional specializations between cerebellar cortical and CN astrocytes. Thus, the CN have very low levels of mRNA expression for aquaporin 4, whereas this astroglial marker is strongly expressed in the white matter and the granular layer. While these differences are particularly striking during the early postnatal period, they may still be recognized in postnatal day 56 animals [145]. Conversely, the glial GABA transporter Gat-3 (Slc6a11) is strongly, and exclusively, expressed in the CN, a fact that has been related to the lack of a GABA-reuptake transporter in PCs [195] (Fig. 10). Lastly, a comparison of the expression patterns of vesicular transporters specific for GABAergic (VIAAT, SLC32A1) and glutamatergic (VGLUT2, SLC17A6) neurons and the glial GABA-transporter Gat-3 (SLC6A1) supports the view that the CN contain an atypically high density of astroglial cells, although the data available in the Allen Brain Atlas are not suitable for detailed stereological counting.

## Physiology of the Cerebellar Nuclei

### Electrophysiology of the Cerebellar Nuclear Neurons

As described in “Classification of Cerebellar Nuclear Cells” and Table 3 above, the CN neurons are currently classified into 5 molecularly and evolutionarily defined classes which, at this level of descriptive granularity, are shared across all CN. The information-processing capabilities, defined by their electrophysiological properties have been reviewed in the past [196], but here we will briefly outline the features relevant for a general understanding of the computational roles in which the CN have been proposed to participate. Notably, the current electrophysiological characterization is largely based on in vitro experiments in juvenile animals without systematic investigation of differences between the CN.

Most CN neurons spike spontaneously [183, 196, 197]. The ionic mechanisms underlying the stable pacemaker capabilities, mainly investigated in a CN subpopulation most likely corresponding to the class-B glutamatergic neurons, are based on persistent non-specific cation currents that continuously drive the neurons’ membrane potential above the spike threshold. Among the voltage- and calcium-dependent potassium channels that support spike repolarization at high frequencies [69, 197–199], differential expression profiles of Kv3.1 and Kv3.3 channels likely underlie the known distinction in firing rates and action potential waveforms between glutamatergic and GABAergic neurons. Broadly speaking, the glutamatergic group (A and B, as well as the glycinergic VN-projecting neurons of the Med) is characterized by short (0.5–1 ms in half-width) action potentials fired at high spontaneous rates (up to or over 100 Hz). The GABAergic group (IO-projecting and putative local interneurons) exhibits broader action potentials (> 1 ms) that cannot be sustained at frequencies beyond a few tens of Hertz. These two “electrophysiological classes” of CN neurons (fast- and slow-spiking) are accompanied by a group of less-studied neurons that maintain a resting membrane potential below the spiking threshold and preferably respond to applied depolarizations with a short-lasting burst of fast action potentials [69], corresponding to the type II glycinergic cells (Table 3). These electrophysiological signatures of CN cell types, obtained in acute slice preparations in juvenile mice, have been largely confirmed by in vivo studies [200–202]. However, as relatively high-frequency spontaneous firing has classically been considered characteristic of CN neurons, it is possible that neuron types with more silent behavior, as well as those with small somata, have been overlooked in many in vivo studies. Finally, all of the CN cell types investigated so far show varying degrees of rebound responsiveness (i.e., enhanced spiking at the offset of a hyperpolarizing or inhibitory input) [184, 203, 204]. Thus, despite classically having been seen as a relay nucleus that simply conveys the results of the cerebellar cortical circuits to downstream targets, it is obvious that the CN neurons can significantly contribute to the cerebellar information processing as a whole. This should be viewed as an invitation for more nucleocentric approaches to be undertaken in cerebellar research, such as the investigation of electrophysiological differences between the different parts of the CN and their unexplored computational capacity.

### Modulation of Nuclear Neuronal Activity by Afferent Inputs

The afferents to the CN originate from the inhibitory PCs, the olivary neurons, other precerebellar neurons, and neuromodulatory systems (see Table 2, Fig. 5). As described above, most CN neurons are spontaneous pacemakers and thus the afferents’ action on the CN is likely best thought of as modulation of the intrinsically generated spikes.

#### Corticonuclear Afferents

As was outlined in **“**Corticonuclear Afferents,” the input from the cerebellar cortex to the CN is conveyed by the GABAergic PC axons. A single PC axon commonly branches within a narrow volume of the CN forming numerous large presynaptic terminals on the somata and proximal dendrites of a handful of glutamatergic projection neurons (classes A and B), each of which is likely contacted by several tens of PCs [205, 206]. The CN somata are engulfed by the PC synaptic terminals and related perineuronal nets [207, 208] delivering inhibitory synaptic activity reflecting the spontaneous high-rate activity of the PCs. The properties of the PC-CN synapses on the glutamatergic projection neurons have been extensively studied and suggest information transfer mechanisms involving a combination of spike rate- and spike timing-based coding [209]. In contrast, the anatomy and physiology of PC axon terminals on non-glutamatergic neurons of the CN have received less attention, even though it is known that the PC synapses on IO-projecting neurons reside on the dendrites rather than the somata [70, 210]. This, together with differences in synaptic short-term dynamics, makes it unlikely that the IO-projecting neurons' spike timing is precisely controlled by the cerebellar cortical input, and suggests that the CN-IO signaling is primarily based on rate-coding principles [191, 211–214].

The massive, convergent GABAergic PC projection from the cerebellar cortex has been seen suggesting that the CN mainly functions as a sign-switching relay element in the cerebellar circuit, especially in a cortico-centric view of the cerebellum (Fig. 1a). In line with this notion, activity patterns of at least some CN neurons indeed mirror the pauses in upstream PC [215–217], amplified by the intrinsic rebound dynamics of CN neurons. Thus, concerted decreases in PC firing rates can drive bursts of CN activity, in turn broadcast as excitation of the various cerebellar target regions [186, 204, 218–220]. Despite the attractiveness of this model where pauses in PC spiking drive activity in cerebellar target structures, the downstream effects likely involve more complex modulations of target network states such as shifting postsynaptic activity patterns between tonic and bursting modes [e.g., 221, 222].

The convergence of cerebellar cortical efferents on single CN neurons, together with the high average PC firing rates has been a source of controversy regarding the mode of information transfer between the cerebellar cortex and the CN. Despite numerous morphological and molecular features of the PC-CN synapses that support reliable high-frequency synaptic transmission [223–225], it is not evident that CN neurons are able to accurately convey information on individual simple-spike timings unless they are perfectly synchronized [205]. Instead, the simple spikes might mainly modulate the average CN firing rate rather than precise spike timing. In contrast, the IO-induced complex spikes could more readily induce distinct pause-rebound sequences in the CN neurons due to intrinsic synchronization properties among groups of PCs [202]. Nevertheless, predictions of CN activity and thus cerebellar output is challenging even in the context of behaviors for which the PC activity has been thoroughly investigated.

#### Olivonuclear Afferents

The glutamatergic olivocerebellar afferents to the CN also project to the PCs as CFs [43, 85]. In the CN, they terminate within the boundaries of olivo-cortico-nuclear loops as discussed in “Efferent Connections of the Cerebellar Nuclei” on cerebellar modules (see Fig. 7), targeting dendrites of at least some of the glutamatergic neurons as well as the IO-projecting GABAergic neurons [42, 87]. It should be noted that neither the presence of IO-originating axon terminals on other CN neuron classes nor the possible differences between Class-A and Class-B glutamatergic neurons has been thoroughly investigated (see Table 3).

The putative distal dendritic localization of the excitatory IO terminals contrasts with the somatic aggregation of inhibitory PC terminals. The low average firing frequency of olivary neurons (approx. 1 Hz) [226] has raised questions on the impact that the olivary input could possibly have on the intrinsically active CN neurons’ spiking. Specifically, it has been argued that the IO input to (distal) CN dendrites in a given olivo-cortico-nuclear micromodule would be masked by the arrival of a near-simultaneous burst of inhibitory synaptic activity following CF-evoked complex spikes in the PCs [e.g., 204, 219, 227, 228]. Yet, this “hidden” short-latency excitation by olivary axons may modulate rebound spiking of CN neurons [204]. Furthermore, even though the Class-A and Class-B glutamatergic neurons display spontaneous high-frequency spiking that might not be much modified by such a slow input, the IO-projecting CN neurons, as well as the cerebellar cortex-projecting glycinergic neurons (type 2 glycinergic neurons) are much less active “at rest.” This might render them more sensitive to IO-originating input. Nevertheless, direct excitation of glutamatergic CN neurons by IO axon stimulation has been demonstrated both in vitro and in vivo [201, 229–231], calling for further investigation of the physiological significance of this pathway in terms of subcellular localization, development [232], and plasticity [229].

#### “Mossy Fiber” Afferents

The non-IO-originating glutamatergic afferent inputs are commonly lumped under the label of “mossy fiber CN inputs,” as many (but not all) of the MF axons providing the cerebellar cortex with multimodal and dense representation of the external and internal states of the world, branch and also terminate in the CN (but see “CN Connections of Mossy Fibers”). For brevity, in this section, we refer to the non-IO-originating glutamatergic afferents as “MF”.

However, we remind the reader that (1) not all cerebellar cortical MFs have been shown to project to the CN and (2) some precerebellar nuclei do not send afferents into the cerebellar cortex in the adult (see Table 2) [65]. Indeed, some connections, such as the direct cerebro-cerebellar projections described in neonate kittens may be lost over time [97, 233], or in contrast, pontine connections to the CN may develop further in adulthood, especially during learning [234, 235], thereby underscoring the importance of these nuclear connections.

To date, no systematic investigations of the differences and commonalities among the MF afferents have been conducted, even though features of even single-source axonal projections are known to vary substantially [57, 67, 236]. Physiology of only the pontine nuclei afferents to the CN [234] has been specifically investigated, and the main body of current knowledge on the MF afferents is derived from experiments obtained with non-discriminating electrical stimulation in vitro [such as 237]. Notably, such studies often cannot distinguish MF responses from those originating from CF branches or local glutamatergic axons.

Nevertheless, the role of these MF inputs in determining CN activity has received significantly more attention than those of the olivocerebellar projections, most likely due to the abundance of the pathways. Stimulation of brain regions providing MFs, as well as sensory stimulation of various body parts has been shown to increase the spike rate of at least some CN neurons [238–241]. Interestingly, these synaptic connections have been shown to be resistant to classic Hebbian plasticity-evoking protocols. Instead, MF-CN synapses undergo long-term potentiation only when input bursts are paired with a delayed inhibition-excitation sequence. The time course of this phenomenon could have physiological significance in a behavioral context in which a transient increase in PC activity, driven by activation of the MF-parallel fiber pathway, would be followed by a pause. This, in turn, would drive an inhibition-rebound sequence in CN neurons [96, 237, 242].

Finally, since evidence has emerged showing terminals of a MF pathway expanding within the CN when the animal undergoes an eyeblink conditioning protocol [234], the MF-CN synapse has gained interest as a possible key site of plasticity involved in cerebellar learning  [243, 245–248]. The scarcity of direct experimental investigations of MF-CN plasticity in living animals limits our ability to draw strong conclusions regarding the role of MFs in shaping cerebellar output. However, it appears evident that the rich sensory and motor reality that modulates cerebellar cortical dynamics is also directly available to the CN, underlining the capacity of the CN to encode behavioral trajectories [249–251].

### Downstream Actions of Cerebellar Efferents

#### Nucleo-olivary Efferents

The CN efferent projection that targets the IO is usually presumed to play a key role in the dynamics related to complex spike-related plasticity processes in the cerebellar cortex [226, 252, 253]. The CN axons branch extensively in the contralateral IO [150, 151, 157], making large numbers of synaptic connections on numerous IO neurons clustered within the olivocerebellar micromodule (see **“**Cerebellar Modules”). The synaptic organization of the sparser ipsilateral pathway remains unclear. The GABAergic nature of the nucleo-olivary projection suggests an inhibitory function in the sense that the probability of IO spiking and thereby cerebellar complex spike occurrence should be reduced by activation of the nucleo-olivary neurons. As PCs inhibit nucleo-olivary cells, they could modulate their own activity by means of the tri-synaptic PC-CN-IO feedback loop [254]. However, this interpretation needs to be amended to account for the specific arrangement of CN axons in the IO, terminating in the vicinity of gap junctions that are the only known means for interneuronal communication in the IO [255, 256]. Indeed, the influence of CN activity in the IO likely extends beyond a simple inhibition to the domain of network synchronicity modulation [153, 159, 257]. Conductances activated by GABA release from the nucleo-olivary axons lead to shunting of the gap junction currents and thereby modulating the strength of signaling among IO neurons and the composition of synchronously active IO neuronal clusters [153, 258, 259]. More investigation is necessary [212, 214], but the slow intrinsic spike generation in IO-projecting CN neurons, the asynchronous transmission properties of the CN-IO synapse [260], as well as the unusual action potential generation mechanisms in the IO [261] make it unlikely that the cerebellar IO-projecting neurons could adjust IO spiking with millisecond precision. Nevertheless, the CN-IO pathway is poised to control coherence within and between functional micromodules [258] in addition to the simple modulation of the excitability of IO neurons.

#### Nucleocortical Efferents

While nucleocortical projections have been described in classic literature [262], it only recently became evident that these connections originate from both excitatory and inhibitory CN neurons, and the inhibitory nucleocortical projection is still not incorporated into the common discourse of overall cerebellar circuitry despite being functionally described in mice [69, 175]. Axons of type 2 glycinergic CN neurons (Table 3) project into the cerebellar cortex where they form large synaptic terminals on the somata and dendrites of the purely GABAergic (as opposed to mixed GABA-glycinergic) subpopulation of cerebellar Golgi cells. These Golgi cells in turn modulate the excitability of the cerebellar granule cells and are proposed to restrict the temporal window for afferent input integration into the parallel fiber-PC pathway. As was mentioned previously (“Electrophysiology of the Cerebellar Nuclear Neurons”), the inhibitory neurons of the CN projecting to the cerebellar cortex are not spontaneously active in vitro but show a preference for fast burst spiking upon depolarization. Activation of such a burst in type 2 glycinergic neurons could broaden the time window for synaptic integration in cerebellar granular layer [263, 264]. It is possible these nucleocortical neurons with strong burst-fire behavior could be efficiently activated by the low-rate activity in olivary projections to the CN, thereby allowing modulation of the specificity of the sensory information that reaches a particular olivocerebellar module.

In contrast to the inhibitory nucleocortical projection, the existence of glutamatergic nucleocortical afferents has been much more widely acknowledged [171, 173, 174, 262,265–267]. It is currently unclear whether there are differences in cerebellar cortical targeting between Class-A and Class-B glutamatergic projection neurons, but evidence from the mouse Lat suggests that both do project to the cerebellar cortex [35]. Functionally, the excitatory nucleocortical pathway seems to play a significant role in modulating the cerebellar cortical circuit dynamics. Considering that activity patterns in the glutamatergic projection neurons likely encode kinematic features of ongoing or planned movements, their projections to the cerebellar granule cell layer can provide an amplifying signal supporting cerebellar computation [174].

While the functional roles of the nucleocortical projections are in need of investigation in terms of anatomical and molecular diversity as well as commonalities of their computational roles across cerebellar striped modules, they are an undeniably prolific signaling pathway that must be taken into consideration in any model of mammalian cerebellar function.

#### Excitatory Extracerebellar Efferents

As was described above (“Efferent Connections of the Cerebellar Nuclei”), the diversity of glutamatergic projections from the CN to various extracerebellar target areas is vast, rendering futile any attempt to comprehensively review their function or morphology. The full extent of the diversity of brain regions that receive direct cerebellar inputs has only recently become recognized, and for the majority of post-cerebellar targets, no detailed descriptions are available beyond mesoscale density estimations [35, 268]. Among the multitude of known glutamatergic extracerebellar target regions, the synaptic function and organization is probably the best studied for thalamic [147, 222,269–272], ventral tegmental area[273] and rubral [274, 275] projections. In these regions, glutamatergic CN axons form synaptic terminals on target neurons' proximal dendrites and/or somata, and synaptic transmission has been shown capable of following axonal stimulation at least up to several tens of Hz without significant depression.

When contemplating the function of these excitatory efferent CN connections, it is not only their diversity that is striking, but also their wide divergence. Single fibers may collateralize to a selection of diencephalic, mesencephalic, pontine, medullary as well as spinal cord levels, implying that the same CN signals are distributed to several organizational levels [35, 149, 166, 168]. Presently not much information is available on the selection and number of target areas of individual fibers originating from a different modular origin and typology (Class-A or Class-B) and the organizational differences that have been noted clearly demand further study [35, 168]. In addition, it will be interesting to speculate how the cerebellar output organization builds on evolutionary older systems (see “Evolutionary Origins of the Cerebellar Nuclei”).

Available physiological evidence shows that the spiking patterns of CN excitatory projection neurons can be reliably transmitted to the target neurons. The ultimate behavioral consequences of activity in the extracerebellar efferent pathways obviously depend on the identities, connectivities, and intrinsic properties of the target neurons that remain to be identified for most regions. Many of them are involved in reciprocal communication pathways with the CN [151, 178, 276, 277], implying that the full significance of CN activity in behavior is unlikely to be elucidated without a holistic, multi-regional experimental approach. Finally, while some evidence has occasionally been reported in the past [e.g., 274], the possible non-glutamatergic cerebellar signaling pathways beyond the IO have only very recently been seriously considered [151]. We expect that the increasingly wide availability of genetically encoded tracing and activity manipulation tools will lead to a substantial expansion of our understanding of the significance of cerebellar signaling in coordinating animal behavior.

### Summary of Cerebellar Nuclear Function: It Integrates, but What Does It Communicate?

**Fig. 11:**
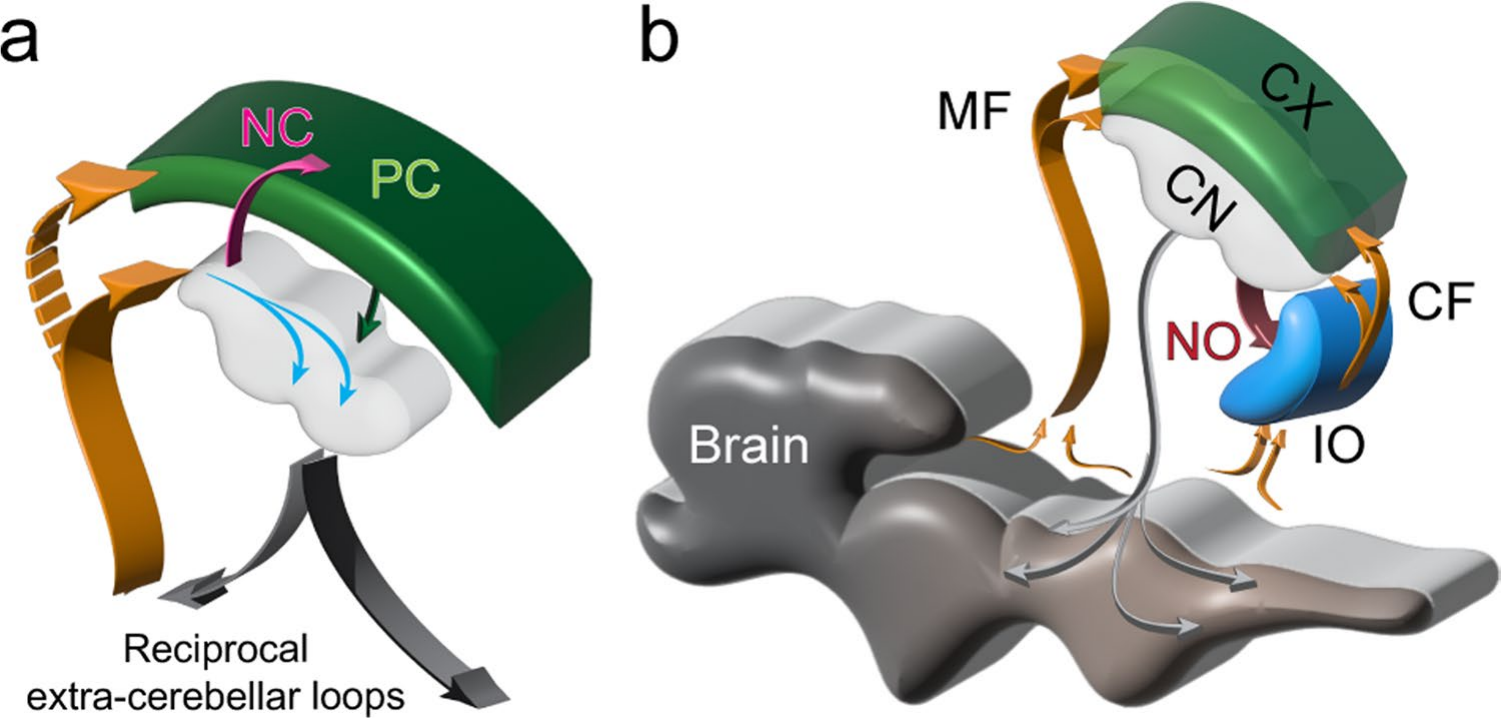
Schematic depiction of the conceptual differences between an approach that considers the cerebellar cortex and the nuclei as independent functional units (**a**) and a view of cerebellar computation where information processing is not segmented into “cortical” and “nuclear” parts (**b**). The latter scheme is a natural extension of the nucleocentric view promoted in this review. From the viewpoint of the whole brain, the cerebellar system appears as a unified and modular computational system. CX, cerebellar cortex; CN, cerebellar nuclei; NC, nucleocortical pathway; MF, mossy fibers; CF, climbing fibers; IO, inferior olive; NO, nucleo-olivary fibers (cf. Figure 1)

Recognition of the richness of CN cell diversity and functional dynamics has been accompanied by a gradual evolution in the prevailing views of its roles in cerebellar computation (Fig. 1 and Fig. 11). The naive concept of the CN as a “simple” relay station that (inversely) forwards signals computed in the cerebellar cortex, possibly modified by “MF collateral” afferents (as depicted in Fig. 1a), is now increasingly seen as lacking in depth. At the very least, it must be complemented by the fact that excluding moments when the cerebellar cortical circuits dramatically synchronize their activity (such as during discrete learning events), the “cerebellar output” is constructed from interactions between intrinsic activity dynamics of CN neurons and afferent inputs, potentially modulated by average PC firing levels (Fig. 1b). However, as we begin to recognize the extent and significance of excitatory and inhibitory nucleocortical projections linking the CN and cerebellar cortical regions [174, 175, 267], it is becoming increasingly challenging to conceptualize functions of CN and the cerebellar cortex in isolation from each other. When seeking to elucidate cerebellar function in the future, there is no choice but to adopt a holistic circuit research approach, in which the structure and function of all of the three components of this system—the CN, the cerebellar cortex, and the IO—are understood to form a unified computing network (Fig. 11a, b) composed of numerous modules (Fig. 7).

What do the CN do to afferent information? Clearly, their function is related to the integration of afferent information streams. However, the term “integration” conveys little insight into the computational and behavioral significance of this operation or into which aspects of the sensory and executive signals are distilled into the cerebellar output. Furthermore, the fact that the result of the cerebellar computation is broadcasted through the narrow bottleneck of a small number of projection neurons suggests that numerous brain regions in both the motor and non-motor systems receiving these signals must extract the information they need from a possibly multiplexed communication channel. Regrettably, this review does not have the space to delve into the communication-theoretic aspects of CN function, but it seems likely that the “meaning” of cerebellar computation is constructed within the circuits linked by reciprocal cerebellofugal and cerebellopetal connections [e.g., 278, see also 279]. Thus, not only should future research into cerebellar circuitry emphasize both the CN and the cerebellar cortex but also investigate the reciprocally connected structures, preferably beyond mesoscale connectomic and dynamic correlations.

Finally, it must be recalled that “nothing in biology makes sense except in the light of evolution [and development]” [280], and in this spirit, we now turn to reviewing current knowledge on these aspects of the CN.

## Development of the Cerebellar Nuclei

Many studies of CN development of the last four decades can be traced back to the seminal work of Altman and Bayer [281–283]. A summary of, and references to, the primarily observational work published before 1940 may be found in Dow [284]. Other studies of outstanding historical interest include those of Rüdeberg [285], Taber Pierce [286], Goffinet [287], and, for human material, the studies by Müller and O’Rahilly [288]. More recently, there is an excellent review of the development of the CN by Elsen *et al*. [289].

### Origins and Birthdating of Neurons in the Cerebellar Nuclei

From the birthdating studies in the rat by Altman and Bayer [summarized in 290] and in the mouse by Miale and Sidman [291] and Taber-Pierce [286] came the mistaken impression that all CN cells originate from a single proliferative zone. This view is no longer tenable. In fact, the neurons of the CN emerge, as do the cells of the cerebellar cortex, from the two proliferative zones of rhombomere 1 of the rhombencephalon: the ventricular zone (VZ) above the fourth ventricle, and the rhombic lip (RL) that defines the boundary between neural precursors of the neuroepithelium and the non-neural ventricular roof plate. There is also evidence of a mesencephalic contribution to the CN [292–294] as a subset of α-SYNUCLEIN^+^ /OTX2 CN neurons seems to originate from the mesencephalon and cross the isthmus toward the rostral end of the nuclear transitory zone. While confirmation using fate mapping is needed, immunostaining for OTX2 and the P75 neurotrophin receptor has been interpreted that this population is derived from the neural crest. This putative mesencephalic/neural crest contingent warrants further studies to identify its role in the formation of the CN. The present review focuses on the contributions of the RL and VZ.

**Fig. 12:**
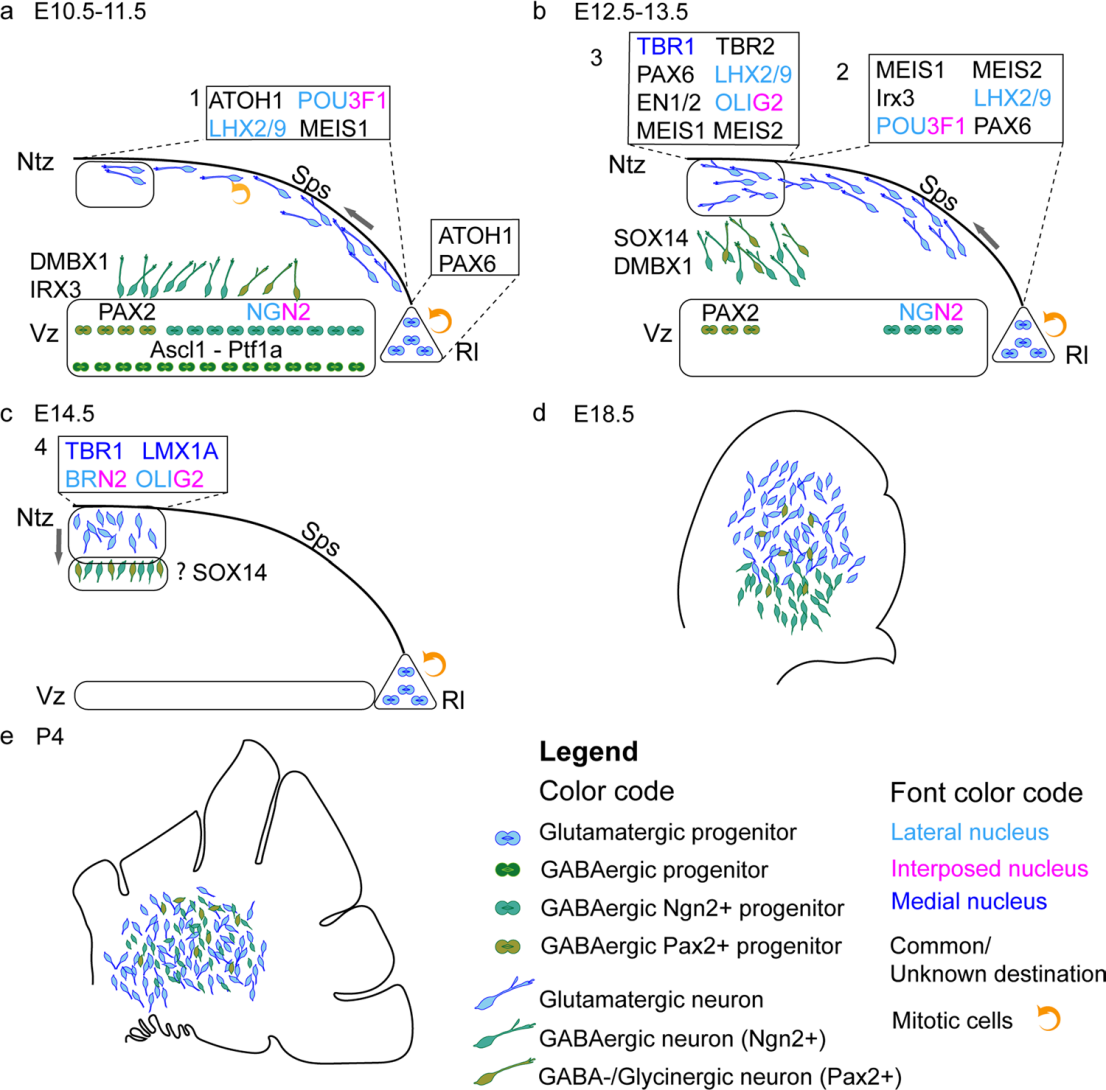
Schematic drawing showing migratory steps of GABAergic and glutamatergic neurons composing the cerebellar nuclei. All sagittal sections are oriented with rostral to the right. **a** Between E10.5 and E11.5 glutamatergic projection neurons (light blue cells) originate from the ATOH1 ^+^ and PAX6 ^+^ progenitors (light blue spheroids) localized in the Rhombic Lip (Rl, Triangular shape). They migrate apposed to the pia mater, forming the subpial stream (SPS, curved line) away from the Rl (gray arrow) expressing the genes indicated in Box 1. Few neurons reach the nuclear transitory zone (Ntz). GABAergic neurons differentiate from ASCL1 ^+^ and PTF1A ^+^ common progenitors (green spheroids) in the ventricular zone (Vz, rectangular shape) from which NEUROG2 ^+^ or PAX2 ^+^ transit amplifier populations originate (green spheroids). Postmitotic NEUROG2 ^+^ or PAX2 ^+^ GABAergic (green cells) neurons leave the Vz expressing IRX3 or Dmbx1. **b** Between E12.5 and E13.5 glutamatergic projection neurons continue their migration expressing several markers (Box 2) and start to reach the Ntz, where they express other markers (Box 3). GABAergic neurons move towards the Ntz and express SOX14 and DMBX1 while IRX3 is downregulated. Neurogenesis of NEUROG2 ^+^ and PAX2 ^+^ neurons appears to continue until E12.25-E12.5. **c** By E14.5 all the prospective glutamatergic CN neurons are located in the Ntz surrounded by a GABAergic population, where they further mature and prepare to descend into their final position in the central mass (grey arrows). **d** By E18.5 all the neurons are in the central mass, occupying different territories: glutamatergic neurons localize dorsally while NEUROG2 ^+^ GABAergic neurons are in ventral and lateral positions. A few PAX2 ^+^ GABAergic neurons are intermingled in the neuronal mass, while the prospective PAX2 ^+^ GABAergic interneurons surround the cerebellar nuclei mass. **e** By P4, the GABAergic and glutamatergic neurons continue small movements to reach their terminal destination. Color fonts of expressed molecules during development indicate the known destination of the cells. The numbers of cells illustrated do not reflect the actual cell numbers, which are mostly unknown

In the light of fate mapping studies that focused on cells that emerged from the *Ptf1a* [295] and *Atoh1* [296, 297] cell lineages, the PTF1A^+^ VZ and the ATOH1^+^ RL were identified as progenitor zones for the inhibitory (GABAergic and/or glycinergic) and excitatory (glutaminergic) cells in the CN, respectively (Fig. 12a). These molecules not only define the two lineages but are critical to the survival of CN cells. PTF1A is required for the presence of inhibitory cells including the GABAergic cells of the CN [295]. The PTF1A^+^ VZ is finely compartmentalized, displaying two VZ microdomains positive for the *Pax2* transcript at embryonic day 12.5 (E12.5; dates refer to mice unless noted otherwise) of gestation that abut those labeled by *Neurog2* and *Neurog1* [298]. How these domains relate to the generation of the CN is not clearly understood. Analysis of a mouse knock-in line expressing Cre recombinase under the control of *Neurog2* [299] demonstrated that between E11.25 and E12.25 NEUROG2 ^+^ progenitors give rise to GABAergic CN projection neurons (Table 3)—the presumptive nucleo-olivary neurons (Fig. 12a, b). In a similar manner, ATOH1 is required in the generation of RL-derived CN neurons as they are found missing in an *Atoh1*-null mutant [296, 300]. However, recent studies of molecules expressed early in cerebellar development indicate that cells upstream of PTF1A and ATOH1 expression comprise lineages that contribute to both inhibitory and excitatory cell types. SOX2 and NOTCH expression appear to be two key players in the early acquisition of cell phenotype in the cerebellar cortex but whether this upstream relationship also applies to the cells of the CN remains to be determined [301, 302].

Much of what we know about the birthdates of CN neurons (i.e., the time of their terminal cell division) comes from tritiated-thymidine studies [286, 290, 291]. These early studies established that large neurons of the CN are the first neurons of the cerebellum to be born, arising, in mice, around E10 [286]. These cells are most likely synonymous with the Class-A and Class-B glutamatergic projection neurons of the CN (Table 3). This conclusion is supported by the more recent observation that TBR1 ^+^ precursors of glutamatergic CN neurons go through their last mitosis “mainly between E10.5 and 12.5” [303]. Similarly, the IO-projecting GABAergic neurons complete their final mitosis between E10.5 and 11.5 [160]. The birthdates and origin of the glycinergic projection neurons identified by Bagnall *et al*. [163] in the Med have yet to be established. Previous studies that examined the *Atoh1* lineage fate mapping by using histochemistry have not observed SLC6A5 ^+^ (i.e., GLYT2 ^+^) cells, suggesting that the majority of glycinergic CN neurons have an origin outside the RL [304]. However, recent evidence based on Atoh1-cre x Ai14 mice, in situ hybridization, and single-nucleus RNAseq shows that the Bagnall *et al*. glycinergic projection neurons are derived from the *Atoh1* lineage, and share extensive molecular similarity with Class-B glutamatergic projection neurons [35], suggesting that these cells also share a developmental origin with the glutamatergic neurons in the early-born cohort. Thus, these neurons defy the general presumption that all GABAergic and glycinergic cerebellar neurons originate in the classically defined VZ.

From the studies of Taber Pierce [286] and Miale and Sidman [291] in mice, and Leto *et al*. in rats [305], we know that in rodents, neurogenesis in the CN persists into the first postnatal week. Generally, the late-born neurons are smaller than the early-born neurons, and they have been tentatively identified as inhibitory interneurons (likely the Type 1 and Type 2 glycinergic neurons of Table 3); alternatively, they have often been referred to as GABAergic interneurons. As should be apparent from the description and classification of CN neurons given above, more recent findings (based upon transmitter phenotype, see [35], and for nucleocortical projection, see [69, 175]; see also “Nucleocortical Efferents” above) clearly indicate that these cells are more heterogeneous than is suggested by their traditional designation as GABAergic interneurons. In fact, in mice most of them are also glycinergic [35]. In humans, about 50% of this population is glycinergic and GABAergic [35]. With respect to this dual-transmitter phenotype (GABAergic and/or glycinergic), they resemble the inhibitory Golgi interneurons resident in the granular layer [306]. One subset of the CN GABAergic/glycinergic cells also projects to the cerebellar cortex (see above, “Nucleocortical Efferents”) but we do not know how these neurons—which were originally lumped with CN inhibitory interneurons—are developmentally related to truly local CN neurons, nor whether the two types may be differentiated based on their birthdates.

It is known that the type 1 and 2 glycinergic neurons of the CN (Table 3) have a developmental history different from those of classical projection neurons. First, they belong to the *Ptf1a* lineage, and secondly they also transiently express ASCL1 [307, 308]. They migrate from the VZ and continue to proliferate while in transit through the nascent prospective white matter, as do the precursors of cerebellar inhibitory interneurons [305, 309–312]. They acquire their eventual positional and neurochemical fates through local, but currently unidentified, instructive cues, and they settle within the CN and cerebellar cortex following an inside-out progression—that is, first in the CN, then in the cerebellar cortical granular layer, and lastly, in the molecular layer [305, 311, for older references and a review, see 313]. This mechanism of cell diversification appears quite different from that in other CNS regions, such as the cerebral cortex where the repertoire of inhibitory interneurons is produced by recruiting precursors from quite different origins [314].

Inhibitory interneuron precursors of the CN, like their cerebellar cortical brethren, also express PAX2 (also express PAX2 [309]; see also supplementary data to reference [35] at https://github.com/justuskebschull/CNcode_final). If CN inhibitory interneurons follow the same rule for PAX2 expression as cerebellar cortical inhibitory interneurons, initial PAX2 expression occurs around the time of their final mitosis [310]. What is known, however, is that at least a subset of mature CN inhibitory neurons maintains PAX2 expression in the adult [35].

While cells of the cerebellar cortex have spatiotemporal neurogenetic gradients [138,315–317], the cells of the CN had not been found to be spatially allocated based upon birthdate. The inability to identify neurogenetic gradients in the CN population was first noted by Taber Pierce in the mouse [286] and largely confirmed in the rat [282] and monkey [318]. Such a gradient may have been obscured in these earlier studies due to the inability to distinguish specific cells (e.g., by genetic inducible fate mapping). More recently, *Wnt1* fate mapping in mice has indicated that a temporal neurogenetic gradient exists in the CN in a lateral (early) to medial (late) manner for the glutamatergic cells of the CN [319]. The same appears to be the case from birthdating studies in the chick [320]. Further unpublished data obtained by taking advantage of a genetic inducible fate mapping performed on transgenic mice expressing an inducible form of the Cre recombinase under the control of an *Atoh1* enhancer [297] support the notion that progenitors fated to occupy the Lat are specified earlier than those bound for the Med (Casoni *et al*., in preparation).

### Initial Migration of Future Cerebellar Nuclear Neurons

As mentioned above, the cells that give rise to the glutamatergic projection neurons of the CN (Class-A and Class-B in Table 3) derive from ATOH1 ^+^ cells of the RL, which migrate tangentially along the surface of the cerebellar anlage, starting at ~ E10, as a subpial stream (SPS—alternatively termed the rostral RL migratory stream) [281, 296] and reach the nuclear transitory zone (NTZ) as early as E11.5 [296, 297] (Fig. 12a). As the CN neuronal progenitors are entering the SPS, ATOH1 expression is dynamically downregulated, and expression of PAX6 (E13.5) [303] and POU3F1 (E10.5) [321] is initiated (Fig. 12a, b). Upon leaving the SPS to form the NTZ, PAX6 ^+^ cells start to express TBR1 and/or TBR2 and concomitantly become PAX6 immunonegative [303] (Fig. 12b). POU3F1 ^+^ cells in the NTZ and the nascent CN also express BRN2 and/or IRX3 and are thought to give rise to a substantial subset of Lat and Int neurons, as defined by these markers [321] (Fig. 12a,b). Additional markers expressed both in the SPS and nascent NTZ include MEIS1, MEIS2, and LHX2/9 [322] (Fig. 12a, b).

What is known about the cells of the VZ? The early-born neurons, which are the GABAergic IO-projecting neurons, are believed to leave the neuroepithelial niche around E11 and come to occupy a region just ventral to the nascent NTZ. These cells are DMBX1 ^+^ at E11.5 (see [145]) and SOX14 ^+^ around E13.5 (Fig. 12a, b) (Allen Institute for Brain Science and [160]). The route by which they relocate from this region to the CN has not been studied. Possibilities include a local accretion of cells to the appropriate nuclei, their *en masse* descent into the CN (Fig. 12b, c) [see 282, 323], or a combination of the two mechanisms. Indeed, specific populations of GABAergic neurons may have differing transit histories. For example, by E13.5 the SOX14 ^+^ cells born in the neuroepithelium accumulate in a wedge comprising the ventral part of the NTZ [160] (Fig. 12c). The IRX3 ^+^ CN precursors at early stages in development (E10.25 and later) have been shown to be in close apposition of radial glial cells, suggesting a mechanism for VZ-born cells to ascend to the NTZ directly or by migration via the pial surface, and then descend [322] (Fig. 12a–c).

**Fig. 13:**
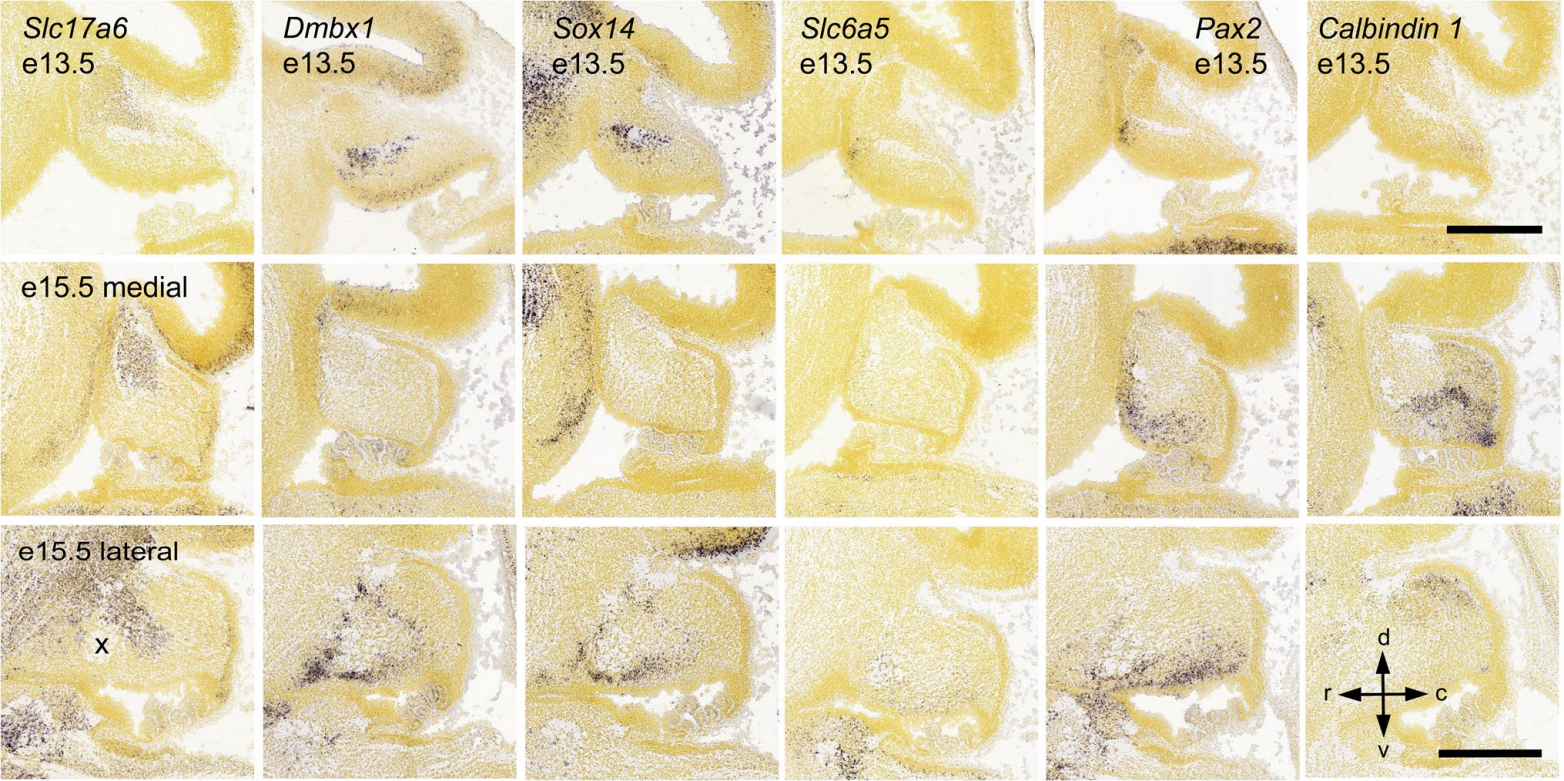
In situ hybridization of molecular markers that allow the identification of excitatory (*Slc17a6*, also known as *Vglut2*) or various inhibitory [*Dmbx1*, *Sox14*, *Slc6a5* (also known as *GlyT2*), *Pax2*] CN neurons at E13.5 and E15.5. For comparison, PCs that express *Calbindin 1* (also known as *Calbindin D28k*) are also shown in the last panel. The top and middle rows show sagittal sections taken from midway between the midline and the lateral border of the cerebellar anlage. The bottom row shows images taken more laterally. At E15.5, the areas occupied by *Sox14*^+^ */Dmbx1*^+^ cells appear to overlap with those occupied by *Pax2*^+^ cells, but not with territories in which excitatory (*Slc17a6*^+^) or *Calbindin1*^+^ Purkinje cells are found. Arrows give orientation (r, rostral, c, caudal, d, dorsal, v, ventral. Scale bars = 0.5 mm (top row for E13.5; bottom row for E15.5) [145]

The means by which the CN cells reach their final location has not been studied. One possibility is that all CN projection neurons (glutamatergic and GABAergic) aggregate in and around the NTZ prior to an inward descent to their terminal location in the CN (Fig. 12c). It has been hypothesized that the simultaneous passing and contact of PCs that are moving dorsally to form the PC plate with the inward movement of the earlier born CN cells from the NTZ might serve as a means for the recognition of CN axons as they travel to the cerebellar cortex, and for the PC axons as they project onto the CN [290, 291]. This, however, does not seem to be the case as closer analysis of these two populations—the descending CN cells and the PCs that are forming the PC plate—shows they avoid one another and do not intermingle. This can be seen in the original description of these cell movements by Altman and Bayer [see figure 3 in 264], and it was later documented in detail by Miyata *et al*. [324]. Lastly, it is also confirmed by the spatial distribution of molecularly identified PC and CN cells as seen in the Allen Brain Atlas [145] (Fig. 13).

In general, the way the CN becomes laid down in a mediolateral row is not well understood. As discussed, preliminary data both in mouse (Casoni, in preparation) and chick (Wingate, *unpublished data*) indicate a birthdating gradient from earliest laterally to youngest excitatory neurons at the midline (see also “Initial Migration of Future Cerebellar Nuclear Neurons”). The implication is that the CN are generated close to the midline and subsequently migrate or are displaced laterally. Intriguingly, in the developmental disorder rhombencephalosynapsis, the agenesis of the cerebellar vermis, the Lat—the earliest born CN—are fused at the midline [reviewed in 325]. This is remarkable as it suggests that during normal development the Lat are displaced laterally by the subsequent arrival of the CN neurons of the more medial nuclei. One clue as to the mechanism comes from studies of LMX1A in the rhombic lip [326]. In the *Lmx1a* null mouse (the mouse mutant *dreher* (*Lmx1a(dr-J)*) there is premature regression of the rhombic lip and hypoplasia of the posterior vermis accompanied by the failure of midline fusion of the cerebellar cortex [327].

How might a failure of midline fusion of the cerebellar cortex be coupled to the failure of lateral displacement of the CN? In *dreher* loss of *Lmx1a* in the RL probably causes to granule cell progenitors leaving the RL prematurely and ending up in ectopic locations [326]. This clearly suggests that in rhombencephalosynapsis defects in the RL may result both in midline fusion defects and a failure to generate CN progenitors.

The mechanisms by which the CN come to occupy their mature mediolateral locations remain speculative. Another possibility is that they are displaced laterally by passive morphogenetic movements. An alternative to the passive displacement hypothesis is that the arrangement of the CN is due to the presence of a covert, pervasive mediolateral gradient. One example might be Joubert syndrome (see “The Cerebellar Nuclei and Joubert Syndrome”). *Zfp423*—a Joubert syndrome gene—codes for a transcription factor and cell cycle regulator [328] expressed in the rhombic lip [329]. In the *Zfp423* mutant, there are EGL defects due to diminished proliferation of granule cell precursors [330] and midline fusion problems associated with profound hypoplasia of the vermis. It is also worth noting that in a *Zfp423* allelic mutation the hindbrain choroid plexus, a derivative of the roof plate, is absent at the level of the midline and rudimental but ciliated in the lateral segments [331]. These data suggest that a medio-lateral gradient of molecules expressed by the roof plate can specify the development of the hindbrain choroid plexus [331, 332]. It is tempting to speculate that the CN are guided by a medio-lateral gradient partially determined by molecules in the midline. Another gene involved in rhombencephalosynapsis, *ZIC2,* is deleted in two rhombencephalosynapsis siblings [333]. ZIC2 belongs to a family of transcription factors and has been shown to be involved in determining gene expression patterns of cerebellar cortical granule cells. *Zic2* is also expressed at E11.5 in the mouse cerebellar primordium (Allen Brain Atlas). Mutations of *ZIC2* might cause an alteration of the gene expression pattern of the CN cell population, leading to an ectopic distribution of these cells. It is easy to imagine that such ectopia of the CN might disturb corticonuclear topography and thus contribute to the CN defective phenotypes in conditions involving defective midline fusion.

Relative to the migration of CN cells, a final comment should be made about the Reelin molecule which when mutated, as in the *reeler* (*Reln*^*rl*^) mutant mouse [334], has been found to be key to the migration of neurons to their correct and final position throughout the brain, including the cerebellum [335]. Of interest, the cells of the NTZ are the first cells to express Reelin in cerebellar development (as early as E13) [335] and thus could have a major impact on the migration of later-developing neurons such as PCs, which remain as clusters of cells in the *reeler* cerebellum rather than the monolayer present in the wild-type cerebellum. The cells that aggregate in the *reeler* NTZ, however, do not seem to be perturbed in their developmental processes [303] although the settling of the CN neurons in the nuclear region appears somewhat abnormal [303, 336]. Likewise, the disruption of cerebellar cortex development in the *scrambler* mutant [337]—a disruption of reelin signaling via mutation of the *disabled* receptor—is not mirrored in the CN and has no obvious effect on normal adult CN anatomy [338].

### Neuronal Subpopulations in and Around the Nuclear Transitory Zone

**Fig. 14:**
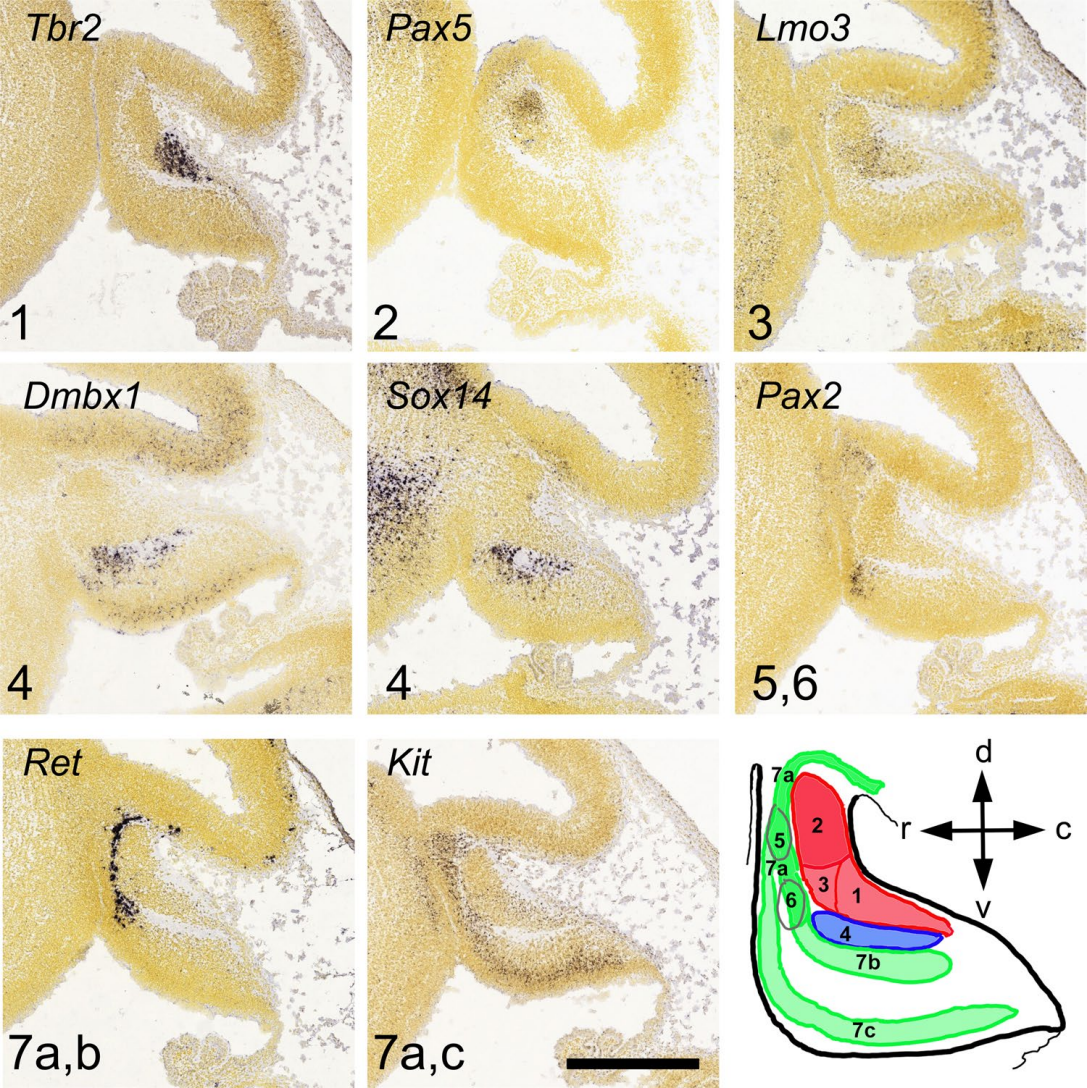
In situ hybridization showing expression of genes that allow the identification of subsets of cells at E13.5 that assemble into the CN. *Tbr2*, *Pax5*, and *Lmo3* mark distinct but apparently partly overlapping sets of cells in the classical NTZ, considered precursors of glutamatergic CN neurons. *Dmbx1* and *Sox14* are markers of inhibitory neurons projecting to the inferior olive. Pax2 ^+^ precursors contribute inhibitory interneurons to the CN. *Ret* and *Kit* are representative of genes expressed in the CN from at least E13.5 onward and into adulthood. Numbers in individual panels refer to the scheme illustrated in the lower right corner. Arrows give orientation (r, rostral, c,caudal, d, dorsal, v, ventral. Scale bar = 0.5 mm [145]

The extended NTZ is formed from 3 sources. The first two sources arise from the ATOH1^+^ expression fields that mark the rostral and caudal boundaries of the cerebellum: the isthmus and the termination of the subpial stream. The third source comes from the accretion of cells from the VZ. This extended NTZ can be visualized based upon cell-specific molecular markers (Fig. 14), examples of which are described below and in Table 4.

**Table 4:** Summary of key genes in the development of the principal/projection neurons of the Glutamatergic and GABAergic/Glycinergic lineages of the CN

Genes with known/suggested role(s) in CN development					
Gene name	Type	Spatial expression in CN	Temporal expression in CN	Expression in other CB cell types	Suggested role
*Atoh1*	Glutamatergic	Rhombic lip [297] and subpial stream [297]	E9.5 in rhombic lip [297], expression ceases as CN cells migrate within SPS [339]	Granule cells progenitors [296, 297], unipolar brush cell progenitors [340]	Generation of glutamatergic CN [296], specification of glutamatergic lineage [339]
*Pax6*	Glutamatergic	Rhombic lip and SPS [303, 341]	E13.5 in cells within SPS [303, 341], expression ceases as CN cells enter NTZ	Granule cells [342], unipolar brush cells [341, 340]	Survival of TBR2^+^ CN [341]
*Tbr1*	Glutamatergic	NTZ, lateral, interposed, and medial CN [303]	E13.5 as cells enter the NTZ [303, 341], remains in lateral and interposed CN until E16.5 [303], medial CN postnatally [303]	Does not express outside CN in the cerebellum	CN morphogenesis/migration [303]
*Tbr2 (Eomes)*	Glutamatergic	NTZ [303]	E13.5 as cells arrived at the NTZ [303, 341]	Unipolar brush cells [340]	Inconclusive [343]
*En1/2*	Glutamatergic	NTZ, medial, interposed, and lateral CN [304]	E12.5 in NTZ, maintained in medial, interposed, and lateral CN post-natally [304, 344]	En1: Purkinje cells, radial glia, granule cells, [344] En2: Purkinje cells, interneurons, unipolar brush cells, granule cells, radial glia [344]	Survival [304]
*Lhx9*	Glutamatergic	NTZ, subpial stream, Lateral CN [320]	E10.5 in subpial stream [326]	Does not express outside CN in the cerebellum in mouse	Overexpression in chick lead to re-specify of axon initial trajectory [320]; TBD in mouse
*Ptf1a*	GABAergic/glycinergic	Ventricular zone [295]	E10.5 in VZ [295]	Purkinje cell progenitors, interneurons, and astrocytes [295]	Generation of GABAergic CN [295], specification of GABAergic lineage [339]
*Sox14*	GABAergic/glycinergic	Nuclear region, Interposed CN, Lateral CN, Nucleus Y of vestibulocerebellar nuclei [160]	E12.5 in nuclear region between the Pax6^+^ CN progenitors and Lhx1/5 + Purkinje cell progenitors [160]	Does not express outside CN in the cerebellum	Migration [160]
Genes with spatial information but no identified function in CN development					
*Brn2 (Pou3f2)*	Both	Interposed CN, Lateral CN [303]		Region directly above VZ of unknown cell identity[344]	Developmental function for CN unknown
*Irx3*	Both	NTZ [341], Interposed CN [321], Lateral CN [321]	E13.5 in NTZ [341]	Not expressed outside CN in the cerebellum	Developmental function for CN unknown
*Pou3f1*	Glutamatergic	Interposed CN, Lateral CN [321]	E10.5 in SpS, maintained in interposed and lateral CN early postnatal [321]	Not expressed outside CN in the cerebellum	Developmental function for CN unknown
*Neurog2*	GABAergic /glycinergic	Lateral CN [299]	E10.75 in NTZ [298]	Purkinje cell progenitors, radial glia [299]	CN phenotype not reported in the KO [299]
*Slc17a6*	Glutamatergic	All nuclei	From E13.5 onward (ABA)	Granule cells; a subset of unipolar brush cells [345]	Developmental function for CN unknown
*Slc6a5*	Glycinergic	Type 1 and 2 glycinergic or mixed glycinergic/GABAergic (inter-) neurons as described in Table 3; Glycinergic/GABAergic projection neurons in the Med [163]	E13.5 in VZ; CN at E18.5; (ABA)	A subset of glycinergic and mixed glycinergic/GABAergic interneurons of the granule cell layer [306]	Developmental function for CN unknown
*Pax2*	GABAergic /glycinergic precursors	Only analyzed by scRNA analysis [35]	So far only documented in adult [35] ABA is equivocal	(Precursors) of cortical inhibitory interneurons	Developmental function for CN unknown
*Pax5*	?	Anterior-medially (ABA)	E13.5 (maybe even earlier) at e15.5, 18.5, p4 very weak signal, potentially in anterior igl (ABA)	Not shown so far	Required for the (early) formation of the cerebellar anlage [346]
*Ret*	Subsets of both glutamatergic and GABAergic/glycinergic cells (Supplementary data [35] at https://github.com/justuskebschull/CNcode_final)	Adult: in all (sub-) nuclei [35, 514]	at least from E11.5 onward (ABA)	Adult: lower ML interneurons; (subset of) granule cell layer interneurons (ABA)	Developmental function for CN unknown
*Kit*	Subsets of both glutamatergic and GABAergic/glycinergic cells (Supplementary data [35] at https://github.com/justuskebschull/CNcode_final)	Adult: in all (sub-) nuclei [35]	from at least E13.5 close to VZ; unambiguously in CN at E18.5;	Inhibitory interneurons of the cb cortex (ABA) [347, 511]	Developmental function for CN unknown
*Dmbx1 (Otx3)*	GABAergic/glycinergic (Supplementary data[35])	NTZ (ABA), medial, interposed and lateral nuclei [35]	E11.5–E15.5 in NTZ	VZ (ABA)	[348]
*Lmo3*	Glutamatergic(Supplementary data [35])	NTZ (ABA), interposed and lateral nuclei [35]	E13–E15.5 in NTZ	Not expressed outside CN in the cerebellum	KO without gross phenotypes [349]

This table includes genes expressed throughout development: from the proliferative zones (E9.5-E12) to their residency in and around the NTZ (E13-E15), to their final positions in the CN (early postnatal to adult). The table summarizes the seminal papers that identify these cells, their patterns of expression, and their suggested functions. Expression data derived from the Allen Brain Atlas (ABA; https://developingmouse.brain-map.org/). A limited number of genes with spatial information but no identified function in CN development are discussed in the text as they currently offer limited insights into the genetics of CN development

The molecular heterogeneity of these cells can be appreciated, at an initial level, by piecing together the data from online in situ [145] and single-cell RNA sequencing (scRNAseq) datasets from circa E13 [350]. The deciphering of a molecular code for these subpopulations may have relevance to their address within the CN. For example, precursors positive for the LIM homeobox transcription factor LHX9 (in mouse) emanate from the RL and migrate along the subpial stream to eventually populate the Lat [320]. Conversely, CN progenitors positive for T-box brain transcription factor 1 (TBR1, first detected at E11.5) are committed to populate the Med, and TBR1 is necessary for the proper migration of these cells [303]. Other transcription factors label precursors in the NTZ that are fated to populate the Med. These include TBR2 [encoded by Eomes, 303], and LMX1A [326], selectively expressed in the cerebellar cortex by the later born posterior zone and nodular zone granule cells [e.g., 351]. Unpublished data (Casoni *et al*., in preparation) suggest that LMX1a ^+^ neurons are more numerous than the TBR1 ^+^ ones. However, it is not clear whether the two populations are partially overlapping. Figure 13 and Fig. 14 illustrate the expression domains of NTZ markers labeling cells that will occupy the Lat + Int or Med CN. The eventual fate for cells identified by some of these markers has been established. Thus, PAX2 expression is specific and characteristic for prospective inhibitory interneurons, both in the cerebellar cortex and nuclei [309, 310, 35]. SOX14 expression identifies prospective GABAergic neurons projecting to the inferior olive [160]; as possibly does DMBX1/OTX3. TBR1, TBR2, and LHX9 are markers (possibly overlapping) for subpopulations of excitatory CN neurons (see Table 4 for reference); and SLC6A5 labels glycinergic neurons.

Interestingly, some developmental markers, including genes that are upregulated in migrating neurons, label a streak of cells that connects the isthmic region to a ventral anterior region of the NTZ. One of these markers, the proto-oncogene RET, remains expressed in the CN at later stages [145]. Another gene of this group, *Tlx3*, is a selector gene that bestows a glutamatergic fate on immature precursors [352] and remains expressed in the CN at E18.5 [145]. Taken together, these observations suggest that the isthmic ATOH1^+^ domain [353] may contribute glutamatergic precursors to the CN.

### Molecules that Distinguish Developmental Stages in the Cerebellar Nuclei

From various lines of evidence including knockout mice, genetic inducible mouse lines, high throughput visualization methods, and microarray analysis, a molecular profile of the development of the various cell types of the cerebellum is emerging. The CN has not escaped these advances. Our understanding of the developmental molecular profile of the CN began with the seminal papers that examined the lineage tracing and knockout of *Atoh1* and *Ptf1a*. These studies found these transcription factors not only mark the progenitors of CN at the early times of cerebellar development but also are required for their generation [295–297] and lineage specification [339].

Further studies of these knockout or lineage-tracing mice have led to the discovery of molecular players downstream of ATOH1 or PTF1A in each lineage. In the glutamatergic lineage, ATOH1 expression in CN progenitors is transient [297] and ceases as the cells migrate within the SPS [321]. CN cells in the SPS express molecules such as LHX2/9, POU3F1, and PAX6 [321, 341, 354]. There appear to be two distinct streams of CN progenitors arising from the RL based on molecular profiles. The first stream arises earlier and is characterized by POU3F1 expression and negative for PAX6 in the SPS; the second stream is evident in the SPS around E13.5 and is characterized by PAX6 positive cells with no POU3F1 expression [303, 321]. The comparison of *Atoh1*-dependent and *Pax6*-dependent transcriptomes, by using microarray analysis, has led to the identification of the role played by PAX6 in the survival of TBR1^+^ glutamatergic CN neurons [341, 355]. The roles played by Pou3f1 and LHX2/9 remain unclear. Despite the important role played by PAX6 in the survival of CN, its expression is transient and ceases as the CN cells enter the NTZ [303, 341] (“Neuronal Subpopulations In and Around the Nuclear Transitory Zone,” above, summarizes some of the molecules selectively expressed by CN neurons in the NTZ).

A current listing of genes known to have a role in CN development is detailed in Table 4. Other molecules, of unknown function but whose expression is present in CN cells during development, are listed at the bottom of Table 4. The analysis of flow-sorted single cells and nuclei in the cerebellum has had the potential to highlight numerous other molecules that are part of the molecular signature of developing CN neurons [350, 356–358]. However, and oddly, the single-cell RNA sequencing (scRNAseq) efforts are currently rather disappointing in delineating the molecular signature of CN cell types in development. This may be due to sampling issues, such as the number of CN cells being rather low compared to the rest of the cerebellum or that the CN was not included in the sample. We know from scRNAseq work [35] that when the focus of the analysis is on the CN (such that CN cells are specifically isolated from the whole cerebellum) there is a rich return of gene profiles that differentiate CN subtypes and uncover an impressive heterogeneity of gene expression patterns. This analysis, however, was from the adult cerebellum, and the expression patterns are quite different from the single-cell developmental work noted above.

**Table 5:** Overview of established or potential CN involvement in human neurological and psychiatric disorders, with the corresponding genetic abnormalities, neuropathological changes, clinical features, and available murine models

*Disorder*	*Etiology*	*Pathology and CN involvement*	*Clinical Features*	*Examples of mouse models (*http://www.informatics.jax.org/allele*)*
*Alzheimer’s disease (AD) *[359]	Buildup of amyloid plaques and tau proteins	Bilateral atrophy in medial temporal regions (hippocampus and entorhinal cortex) and the superior parietal lobe. Within the cerebellum, AD patients show significantly higher levels of cell cycle markers and DNA damage response proteins in the Lat, indicative of cellular stress	AD is the most common neurodegenerative cause of dementia. Deficits include loss of episodic memory for recent events, loss of spatial memory, decreased performance at work, and word-finding difficulties. AD finally progresses toward a state of global dementia. Tremor, ataxia, deficits in speech, language, and motor planning are also observed	The triple-transgenic mouse model of AD (3xTg-AD), harboring two human transgenes causing early onset AD (PS1M146V and APPSwe) and a mutant form of tau (tauP301L), mimics both Aβplaques and tau neurofibrillary tangles, following a regional and temporal involvement homologous to humans. The 3xTg-AD cerebellar nuclei show a gradient of neural loss through the mediolateral axis, with the fastigial nucleus being the most affected area, followed by the interpositus nucleus, and the Lat not being affected. [360, 361]
*Autism spectrum disorders (ASD)* [362, 363]	Heterogeneous: most likely caused by a combination of genetic, epigenetic, and environmental factors during neurodevelopment	Cerebellar vermis hypoplasia, reduction of superior cerebellar peduncle, decreased connectivity of dentatorubrothalamic tract	Heterogeneous spectrum of clinical features affecting social interaction, communication, and behavior	*Itgb3* KO (Itgb3tm1Hyn) [365], bilateral CN hypoplasia [366]; *En2* KO (En2tm1Alj), enlarged superior cerebellar peduncle [367]; *Fmr1* KO (Fmr1tm1Cgr/J) [368] Significantly decreased volume of the interposed and fastigial nuclei [369]
*Dentato-olivary dysplasia with intractable seizures in Infancy [*512*, reviewed in *370*]*	Unknown; likely inherited as an autosomal recessive trait	Malformations of cortical development, pachygyria, polymicrogyria, and lissencephaly, cortical calcifications, attenuation of white matter, enlarged ventricles, hypoplastic corpus callosum, overfolded gyri, and glioneuronal heterotopia The Lat nuclei, appear as solid ovoid structures rather than as the characteristic thin, convoluted band	Hypotonia with frequent seizures from birth and gross developmental delays. Survival is no longer than 3 years	None
*Dentatorubral-* *pallidoluysian atrophy (DRPLA)*	Autosomal dominant neurodegenerative disease. Caused by a GOF mutation (expanded CAG triplet repeat) in the Atrophin 1 (Atn1) gene	Combined degeneration of the dentatorubral and pallidoluysian systems. The suffix “luysian” refers to the subthalamic nucleus, involverìd in the indirect pathway The globus pallidus, especially the lateral segment, and the Lat nuclei show loss of neurons and astrocytosis	Progressive disorder causing involuntary movements, mental and emotional problems, and a decline in cognitive abilities. Neurological signs include myoclonus, seizures, ataxia, choreoathetosis. Other signs include intellectual disability, dementia and psychiatric changes (e.g., delusions)	A transgenic line expressing truncated atrophin (Tg(Prnp-ATN1)150Dbo) [371]
*Friedreich ataxia *[372, 373]	Caused by autosomal recessive mutations in the frataxin (*FXN*) gene, encoding a mitochondrial protein involved in cellular iron homeostasis. The mutations (intronic repeat expansion) significantly reduce the expression of *FXN*	Iron dysregulation leading to progressive neuronal atrophy, mainly involving dorsal root ganglia and spinocerebellar tracts In the Lat, severe neuronal atrophy of glutamatergic projection neurons. PCs are also involved with disruption of synaptic terminals that connect with the Lat (grumose degeneration) [374]; Reduced thickness of retinal nerve fiber layer	Peripheral sensory neuropathy; progressive ataxia and motor disabilities	*Fxn* KO (Fxntm2.1Mkn) [375]; Rosa26-targeted *Fxn1*RNAi (Gt(ROSA)26Sortm1(H1/tetO-RNAi:Fxn)Dhg) [376]
*Joubert syndrome *[377–382]	Autosomal recessive disease. ~ 50% of cases are caused by mutations in genes that encode parts of primary cilia or basal bodies. Cilia sense morphogens like Wnt and Shh during development	Malformation of brainstem structures; cerebellar vermis hypoplasia, dysgenesis or agenesis of the cerebellar vermis; deep posterior interpeduncular fossa; thick and elongated superior cerebellar peduncles Malformation/faulty decussation of scp (molar tooth sign) Fragmentation of the Lat nucleus among other hindbrain changes; bilateral CN hypoplasia [378]	Congenital ataxia, hypotonia, episodic breathing dysregulation, mental retardation, and abnormal eye movements	JS3: *Ahi-/- (Ahi1tm1Jgg)* [383]; JS5: *Cep290tm1.1Jgg* [384]; JS7: *Tmem67tm1Dgen*, [385, 386]; JS17: *Cplane1b2b012Clo* [387]; JS26: *KatnipGt(RRG309)Byg* [388]; *Arl13blox/lox(Arl13btm1.1Tc); NexCre(Neurod6tm1(cre)Kan)*
*Parkinson's disease (PD) *[389–391]	Familial and non-familial forms	Degeneration of dopaminergic neurons of the substantia nigra pars compacta leading to dysfunction of striatopallidal circuits. PET results have highlighted a tremor-related network involving the Lat nuclei [391]	Tremor at rest, rigidity, bradykinesia, postural instability and non-motor signs (fatigue, depression, anxiety, dementia, autonomic dysfunction)	Neurotoxin-based approaches: 6-OHDA, MPTP, rotenone (pesticide), paraquat (herbicide), and maneb (fungicide). Chronic administration of neurotoxins induces models of progressive PD. Genetic or virally induced models are based on monogenic forms of PD, including SNCA, LRRK2, UCH-L1, PRKN, PINK1, and DJ-1, as well as manipulation of dopaminergic transcription factors [reviewed in 513]
*Pontocerebellar hypoplasias, *[393, 394]	A group of autosomal recessive neurodegenerative disorders; defects in tRNA splicing and other spliceosome or pre-mRNA complex cleavage genes	Spinal cord anterior horn cell degeneration Cerebellar hypoplasia and cerebellar and pons atrophy; neuronal loss in basal ganglia, gliosis in the brainstem, gliosis in the basal ganglia; scattered loss of PCs Segmental loss of Lat CN neurons while specific regions of the nucleus are preserved	Severe psychomotor delay and intellectual disability; uniformly fatal early in life	*Ppil1em4Jgg*, *Ppil1em3Jgg*, with a severely reduced cerebral and cerebellar size, [395]; Clp1em2Slac, displaying the loss of type 1B neurons in the Lat [396]

### The Origins of Cerebellar Nuclei Afferent Connectivity

#### The Corticonuclear Projection

The development of the corticonuclear projection from the PCs in the cerebellar cortex to the CN is not well understood. The PCs are born at much the same time as the glutamatergic CN neurons (between E10 to E13—see above). An early horseradish peroxidase study in rat cerebellar slices showed corticonuclear PC projections are present by E18 [~ E16 in mice: 397]. However, PC axonogenesis begins at about E12 [324] and *Pcp2-*tagged PCs project their axons into the CN soon after they are born [398]**.** Indeed, PC axonogenesis must begin soon after their birth as their axons are already observed in the mouse CN and peduncles at E14.5. At this stage, both PCs and CN neurons are still migrating. This indicates that the basic corticonuclear projections are established before the somata are in situ. Subsequently, the physiological maturation of the PC projection extends over the next month or so, on a timetable dependent on their Zebrin II + / − phenotype [217].

#### The “MF Afferents”

In the adult mouse, “MF afferents” terminate as glutamatergic synapses both in the CN and on the dendrites of the granule cells in the cerebellar cortex. As discussed above (**“**Connections of the Cerebellar Nuclei”) the terminal fields of the MF in the cerebellar cortex are highly topographically organized into stripes aligned with overlying PC stripes [reviewed in 72, 129]. The MFs are the earliest afferents to enter the cerebellar anlage, led by trigeminal ganglia-originating axons at E9 [81, 294]. Notably, the first targets of the trigeminal ganglia axons are neurons of the CN and not the PCs of the cerebellar cortex; highlighting the centrality of CN in the organization of the entire cerebellar circuitry. Trigeminal projections to the PCs are first observed a day later. That being said, whether the first cerebellar afferent contacts consistently target the CN—implying a critical role for the CN in the early establishment of cerebellar afferent topography—is speculative. The cerebellar cortical MF that develop later also synapse initially on transient targets as many MF afferents enter the cerebellum at a stage at which the granular layer is not yet present [e.g., reviewed in 399]. Near birth, for example, the MFs that have reached the cerebellar cortex synapse ectopically on the PCs. At least some of them also project to the CN at that time [398, 400]. Subsequently, once postmitotic granule cells begin to migrate ventrally from the external granular layer through the PC layer to form the mature granular layer [reviewed in 351], MF axons detach from the PC somata and synapse with the transiting granule cells. Presumably, the ingrowing growth cones recognize PC and CN subtypes, and thereby guide the formation of the adult striped topography.

#### Afferents from the Inferior Olive

Unlike the “MF” afferents to the CN that originate from locations that can be extremely distal (such as spinocerebellar projections), the IO neurons sending glutamatergic input to the cerebellum develop close to the cerebellum in the caudal RL [82, 401, 402]. Growth cones of nascent CFs enter the cerebellar anlage starting at around E14 [403], shortly after PCs become postmitotic. The modular CF arrangement is evident early, by E15, including targeting different CN [82]. While in adult mice the CF innervation is classically considered to be minor (compared to the cerebellar cortical innervation), this may simply reflect the fact that there are ten times more PC than CN targets. During prenatal development, the CN in fact could function as a major organizer of the olivocortical projections by way of providing growth-signal factors [404]. The developmental signals contributing to the maturation of the IO-CN projection remain to be clarified, hopefully providing additional insights into its elusive functional significance.

### The Early Growth of Cerebellar Nuclear Efferent Axons: Insight from the Avian System

While so much of the function and development of CN has been established in the rodent model system, insights from avian anatomy shed important light on the early stages of the formation of CN axon trajectories. In particular, early studies using DiI applications to the RL showed the distinct morphology of presumptive axons in successive waves of cell production from the RL [405]. The long, leading processes of migrating CN neurons extend out of the cerebellum, guided by Netrins secreted at the ventral midline, long before neurons have completed their migration into the NTZ. These leading processes are not retracted but transform directly into axons.

The first cohorts of cells born form a heterogeneous population of neurons with both ascending and descending projections specified at the RL [320, 406]. However, genetic fate mapping reveals that *Atoh1*-derived (presumptive glutamatergic) cells make only rostral axon projections [405]. This implies that at least two populations of non-glutamatergic, ATOH1-negative CN neurons, which project to the ipsilateral hindbrain, are also generated at the early rhombic lip from progenitors that likely express PTF1A (Wingate *et al*., unpublished observations).

How then are descending glutamatergic axons formed? At the border of the NTZ, the leading processes of CN neurons that will form the medial nucleus in chick make a sharp turn rostral towards the isthmus [405]. These axons then arc across at the isthmus in the uncinate bundle, rostral to the cerebellum, and in doing so make a 180° turn to extend caudally and contralaterally [320].

What drives the specificity of early choices in axon trajectory is unknown, although comparative approaches may reveal some insight. Birds lack a Lat CN, which in mammals is characterized by the expression of *Lhx9* [35, 320, 353], a *LIM-homeodomain* gene that specifies axons targeting the thalamus [407]. Over-expression of *Lhx9* in the chick Med disrupts the orderly pathfinding of axons at the boundary of the NTZ but does not forward-engineer the appearance of a mammalian Lat [320].

Finally, it is notable that the sharply defined early axon trajectories are obscured by the promiscuous spread of axons throughout the brain in the adult, such that the multiple targets of axons of any given CN are largely overlapping [35]. Much of the development of connectivity must therefore take place at later stages and the early, precise scaffold of projections is possibly a remnant of a more ancient program of CN development.

## Evolutionary Origins of the Cerebellar Nuclei

### The Cerebellar Nuclei in Anamniotes

The status of the cerebellum in the most basal, jawless vertebrates (Agnatha) has been debated for over a century [408–412]. All vertebrates, including agnathans (hagfish and lamprey), display a rhombomere 1, bounded rostrally by an FGF ^+^ isthmus and containing PTF1A^+^ VZ progenitors with an ATOH1^+^ RL [412, 413]. However, a classic three-layered cerebellar cortex is absent from both lampreys and hagfish [414] and a recent scRNAseq study of the lamprey brain reveals a complete absence of expression clusters that correspond to either PCs or granule cells [415]. Clearly, the possession of appropriate progenitor lineages within the VZ and the RL is not sufficient to produce a cerebellum.

While lampreys and hagfish lack a cerebellum, they nevertheless have sophisticated cerebellar-like circuitry in the hindbrain for processing signals from the vestibular and lateral line systems. Such cerebellar-like circuits are found throughout the vertebrate lineage, processing a range of sensory information from electrosensation (e.g., the dorsal octavolateral nuclei processing inputs from the ampullae of Lorenzini in sharks and rays; [416]) to sound (e.g., birds and reptiles [417]). Like the true cerebellum, cerebellar-like structures are adaptive filters and possess granule-like cells that project parallel axons that synapse orthogonal to dendrites of PC-like, principal output cells [418]. However, in contrast to the cerebellum, cerebellar-like structures do not receive CF input and their principal (i.e., PC-like) output is excitatory rather than inhibitory. Importantly, the output of these PC-like neurons is not mediated by a structure equivalent to a CN.

The precedent of cerebellar-like circuits in evolution has long raised the question of whether the cerebellum emerged as an expansion of these structures. As Rudolf Nieuwenhuys noted in 1969, the cerebellum might best be described as a “…forward extension and a specialization of a structure already present in the dorsal-most part of the rhombencephalon” [419]. This would suggest that the cerebellum in these clades acts functionally as a “head ganglion” of the proprioceptive system [420] that is less involved in the direct modulation of movement than in the processing of sensory and proprioceptive inputs. This predominantly sensory and proprioceptive role might explain the massive expansion of the cerebellum in some ray-finned fish species that depend heavily on lateral line and electrosensory systems for a wide range of behavior. The gigantocerebellum of weakly electrosensory mormyrid fish [421] is used not only for processing information about its environment in muddy water but also for a wide range of social interactions including courtship.

**Fig. 15:**
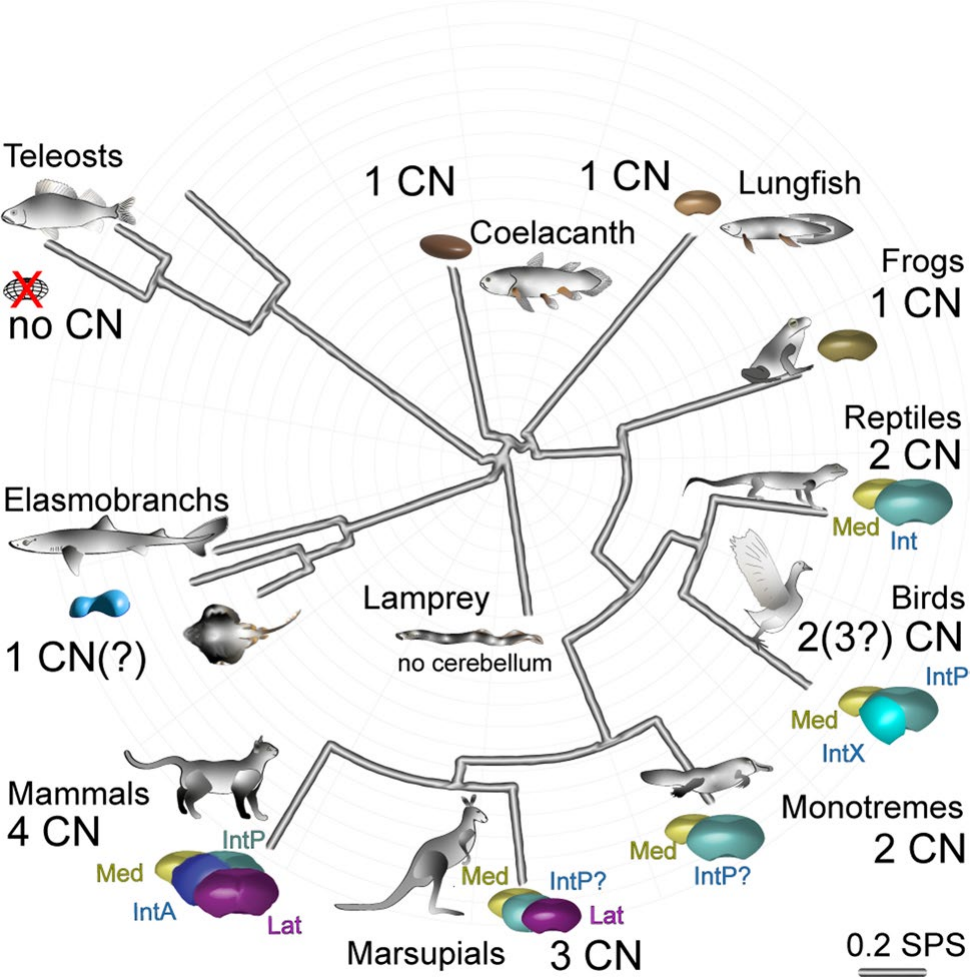
Proposed diversification of the CN in vertebrates, visualizing the appearance of new CN subdivisions during evolution alongside increasing behavioral complexity. Note that in several shark species the CN is clearly divided into two parts, but their functional independence in terms of downstream connectivity has not been examined. SPS, substitutions per site in the dendrogram, reflecting relative amount of genetic changes since the previous branch. Dendrogram based on [130] and [422]

When the cerebellum emerged in jawed vertebrates, there was a distinct divergence in both morphology and circuit arrangement. While basal jawed vertebrates represented by sharks and rays (chondrichthyan) display a distinct CN, the more derived (evolutionarily more recent) ray-finned fish (actinopterygians, including teleosts such as the mormyrid) have none (Fig. 15). This raises the question of whether CN have been lost in ray-finned fish or arose independently on more than one occasion in the vertebrate lineage.

The cerebellum of sharks and rays displays some features reminiscent of cerebellar-like circuits. The cerebellar granule layer is co-extensive with the cerebellar-like structures of the hindbrain (e.g., the dorsal and medial octavolateral nuclei) and cerebellar granule cell axons in rhombomere 1 [411] can extend axons into the dorsal cerebellar “crest” of the hindbrain to activate PC-like cells [418, 423]. However, in contrast to PC-like cells, cerebellar PCs in sharks receive a CF input and project to the CN. This nucleus is organized into distinct medial and lateral compartments, contains both glutamatergic and GABAergic neurons [424], and receives MF input [418]. Efferent projections from the shark CN seem comparable to other vertebrates in breadth and projection patterns [425] and the CN is inhibited by the cerebellar cortex [239]. Yet, GABAergic axon terminations appear to be absent from the shark CN [424].

By contrast, in ray-finned (actinopterygian) fish, including teleosts such as zebrafish and the most basal chondrosteans such as the sturgeon [426], PCs project not to CN but to eurydendroid cells embedded within the cerebellar cortex [427, 428]. Eurydendroid cells derive from both ATOH1 ^+^ and PTF1A ^+^ (OLIG-2 ^+^ sub-type) progenitor pools [429] and are exclusively excitatory [430, 431]. This organization may relate to the origin of most cerebellar cells from a unique, specialized stem cell node at the rostral tip of the cerebellum called the valvulus. The valvulus takes on the production of cerebellum cells from the RL and VZ after early larval stages are complete [432, 433] to constantly supply new cells of all types to the cerebellum, as it grows continuously throughout life. The valvulus replaces multiple progenitor zones with a single source of neurons acting much like an apical meristem in plants.

Answering the question of when CN arose in evolution involves understanding why ray-finned fish have no CN, even at larval stages, when their shark ancestors apparently do. One possibility is that the CN progenitors of the ray-finned fish have been co-opted into a different circuit. The early-born RL-derived populations in ray-finned fish, which are developmentally equivalent to CN, have a non-cerebellar fate in the tegmentum [434]. Such cells are potentially the developmental counterparts of the CN which have been repurposed in actinopterygians. Alternatively, the shark CN may have a developmental origin outside the cerebellum, possibly in the hindbrain or mesencephalon [293, 294], and is hence not a true homolog of those in amniotes [435, 436]. In the former case, the CN have always formed part of the cerebellum but are cryptic (or even lost) in ray-finned fish. In the latter case, neither shark nor teleosts display a true cerebellar-derived CN and their cerebellum would be more clearly “cerebellar-like” in developmental terms. If this were the case, the “first” CN and hence, in a nucleocentric view, the first “true” cerebellum—would emerge in the tetrapod lineage from which amniotes arose. Partial evidence for this comes from the relatively small CN of the lungfish (*Neocertodus forsterii*), first identified morphologically by Holmgren and van der Horst in 1925 [437, 438], which abuts the granular layer and is thus reminiscent of the embryonic NTZ of the tetrapod. Nothing equivalent to the NTZ is apparent in either ray-finned or chondrichthyan fish. The significance of the appearance of the NTZ in sarcopterygians would be in allowing the cerebellum of the future amniote tetrapod, equipped with RL-derived CN, to shift its function away from a proprioceptive and sensory predictive role towards explicit control of motor and cognitive functions via its CN. An important future insight into this fundamental evolutionary question will lie in confirming or refuting the origins of the shark CN as either cerebellar-derived [285] or extracerebellar in origin [435, 436].

### Evolutionary Modular Expansion of the Cerebellar Nuclei: Amniotes

The cerebellum of land-dwelling tetrapods displays a varied number of anatomically distinct CN. An attractive hypothesis is that the progressive subdivisions of the CN into distinct sub-regions occurred in parallel with increasing functional sub-specialization and a diversification of output to different brain regions. The most evident example of this trend is the multiply folded “dentate” nuclei of chimpanzees and humans that serve the pathways connecting expanded cerebellar hemispheres to the massively expanded prefrontal cortex by way of the thalamus. How similar is the composition of each amniote CN: do they represent the iteration of the same basic CN evolutionary module, or the addition of novel subunits with distinct characteristics?

A recent scRNAseq study has gone some way to answering this question. It revealed that the CN across amniotes, such as birds and mammals whose last common ancestors were aquatic, share a remarkable conservation of a modular architecture [35]. Birds and mammals are classically considered to have two and three CN, respectively (see Fig. 15). Each of these CN can be subdivided into smaller, spatially segregated, and cytoarchitecturally distinguishable subdivisions. Kebschull *et al*. [35] show that despite having different numbers of subdivisions, each individual substitution in mice and chickens contains three inhibitory neuron classes and two excitatory neuron classes, as detailed in Table 3. It thus appears that the CN expanded in amniotes by adding additional sub-CN of equivalent cell type structure. Notably, recent evidence from mouse development indicates that the number of excitatory CN neurons controls the number of PCs [304], which in turn controls the number of granule cells [439]. It is therefore tempting to speculate that the evolutionary or developmental emergence of a new cerebellar sub-CN will “automatically” trigger the production of the appropriate amount of cerebellar cortex to go with it (see also “Cerebellar Modules”).

An exception to the stereotyped cell type composition of three inhibitory and two excitatory cell types in each of the CN is seen in humans [35]. While the human Med and Int conform to the canonical composition of amniote CN as described in Table 3, the morphologically distinct Lat appears to have lost one of the two excitatory neuron classes (Class-A, Table 3). However, the human Lat maintains the other excitatory class (Class-B, Table 3) and all three inhibitory classes. Fine-tuning of cellular abundances is hence possible within the theme of modular expansion of the CN.

A parsimonious model for the evolutionary diversification of the CN is the modification of the temporal succession of neuron production at the RL. Neurons are committed [406] to a given fate according to a strict sequence of birth dates within the ATOH1 progenitor pool [296, 297, 353]. This allows for evolutionary innovation by temporal cohort multiplication where novel CN are established by the insertion of a new population of RL-derived, evolutionary module-specific excitatory neurons into a sequence of cell production. This appears to be, for example, the origin of a mammalian Lhx9 + lateral nucleus that is not present in birds [320]. The diversification of RL-derived glutamatergic cells comprises a scaffold for the generation of diverse CN. By contrast, GABAergic CN neurons, generated from PTF1A^+^ cells, are layered onto this template. Such a model is supported by scRNAseq data showing that module-invariant, VZ-derived inhibitory neurons populate both new and old evolutionary modules without changing their adult transcriptomic states [35].

## Disorders of the Cerebellar Nuclei

Because of the importance of the CN as the dominant cerebellar output, diseases and developmental disorders that involve perturbations of the CN are expected to lead to severe consequences. However, due to the tight functional coupling between the CN and the cerebellar cortex, it is difficult to localize cerebellar deficits, and to date, no disorders have been exclusively linked with the CN. However, correlative evidence suggests that the role of the CN in neurologic diseases may have been underestimated. For example, clinicians have long known that various disorders that feature dysmetria, dysarthria, and dysphagia in humans are more disabling when due to lesions in the Lat than when the damage occurs in the cerebellar cortex [440]. Similarly, several spinocerebellar ataxias (SCAs) have been associated with pathologies or dysfunction of the Lat [440]. In addition to these examples, we propose that some defects assigned to the cerebellar cortex may have an important CN component. Recent findings indicate that selective embryonic ablation of excitatory CN neurons leads to a loss of PCs and reduced postnatal growth of the cerebellar cortex [304]. Thus, while PCs also control their own survival, cell autonomously or through paracrine interactions [e.g., 441], the development of the CN influences the survival of their PC partners with repercussions for other cerebellar cell types. Defects in the CN may thus cause problems in the cerebellar cortex, as opposed to being secondary to them. Table 5 summarizes knowledge on established or potential CN involvement in human neurological and psychiatric disorders, some of which are discussed in detail below.

### The Cerebellar Nuclei and Ataxias

The term ataxia defines a set of clinical signs, such as an impairment in the ability to maintain balance during gait, or a loss of trunk, limb, and eye coordination. Moreover, the word ataxias is used to refer to a set of mostly hereditary disorders, characterized by motor incoordination, which is frequently accompanied by degeneration of the cerebellar cytoarchitecture. While many forms of ataxia are caused by malfunction of the cerebellum proper, others involve the proprioceptive tracts. Hence, ataxias may result from either motor or sensory components, and not all ataxic patients display obvious structural changes in the cerebellum. Ataxias have been extensively reviewed in recent years [inter alia 443, 444]. Among inherited ataxias, Friedreich’s ataxia (FA) is the most clearly defined. It is an autosomal recessive degenerative disorder of motor coordination characterized by symptoms and signs that include slowly progressive ataxia of gait and upper limbs, associated with dysarthria and loss of position and vibration sense in lower limbs. FA has its onset between the end of the first decade of life and the beginning of the second one and results in severe disability. FA patients express very low levels of the frataxin (*FXN*) gene, encoding a mitochondrial protein involved in cellular iron homeostasis [372]. The resulting iron dysregulation leads to progressive neuronal atrophy in both the central and peripheral nervous systems. While in FA the spinocerebellar tracts degenerate at the onset of the disease, together with the posterior columns, pyramidal tracts and peripheral nerves, the progression of this disease appears to be affected by selective, presymptomatic loss of large glutamatergic neurons (likely Class-B) in the Lat [373, 374, 445–448]. The degeneration of these projection neurons is accompanied by a ~ 60% reduction of the scp cross-sectional area in FA patients.

Unlike Friedreich’s, the spinocerebellar ataxias (SCAs) are a genetically heterogeneous group of autosomal dominantly inherited progressive disorders, the clinical hallmark of which is loss of balance and coordination accompanied by slurred speech; their onset is most often in adult life [449]. The genetic changes underlying SCAs comprise repeat expansions in coding and non-coding regions, as well as point mutations and duplications (for details, see Online Mendelian Inheritance in Man, omim.org). At least three forms of spinocerebellar ataxia (SCA1, SCA3, and SCA20) involve the CN. SCA1 is caused by the expansion of a CAG repeat located in the coding region of the disease-causing gene *ATX1*. This mutation results in the production of a mutant protein ataxin-1, with an extended polyglutamine stretch. The mutant protein builds up in the cell nucleus disrupting gene transcription, ultimately leading to cell death. Its most prominent diagnostic pathological feature is olivopontocerebellar atrophy, with neurodegeneration predominantly affecting PCs and the Lat nuclei of the cerebellum [450]. In addition to SCA1, SCA-2, SCA-3/Machado-Joseph disease, SCA-6, SCA-7, and SCA-17, all of which are “polyglutamine diseases,” exhibit changes that affect a reciprocal circuitry between the cerebellar cortex, the Lat, and the IO, unlike other forms of SCA in which no clear signs of CN involvement has been detected. Indeed, while the Lat displays degeneration in SCA-3/MJD, the cerebellar cortex and the inferior olivary nuclei remain largely unaffected in this disorder [451]. In the case of SCA-3, it was also reported that large, presumably glutamatergic, neurons (likely Class-B) in the Lat are selectively destroyed [440]. Another remarkable observation is the finding of Lat nuclei calcifications in SCA20, resulting in a low signal on brain MRI sequences [452].

Finally, dentatorubral-pallidoluysian atrophy, caused by a CAG repeat expansion in the *ATN1* gene, is a rare autosomal dominant disorder characterized by myoclonus, epilepsy, ataxia, and dementia. It affects a circuitry involving the Lat nucleus, red nucleus, globus pallidus, and subthalamic (Luysian) nucleus, which display neuronal intranuclear inclusions, variable neuronal loss, and astrocytosis [453].

### The Cerebellar Nuclei and Joubert Syndrome

Joubert syndrome (JS) and JS-related disorders (JSRDs) are autosomal recessive conditions that belong to a class of diseases called ciliopathies [454]. JSRDs are caused by mutations in over 30 different genes involved in the structural and functional regulation of the primary cilium, a critical sensor protruding from the plasma membrane of neural progenitors and mature neurons, which acts as a hub to detect and transduce a variety of extracellular signals, including morphogens and mitogens [455]. Neural progenitors express a cilium on their surface and use it during cell division and cell fate specification [456].

JS patients suffer from cerebellar ataxia and other neurological deficits [454, 457]. Patients affected by JSRDs display a distinctive defect in cerebellar and brainstem ontogenesis observed in axial MRI sections known as the “molar tooth sign,” consisting of a malformed and elongated scp, in many cases accompanied by an expansion of the fourth ventricle [377]. This radiological abnormality results from a combination of hypoplasia of the cerebellar vermis and defective targeting of the scp, which fails to properly decussate [457, 458]. While the molecular mechanisms causing scp misrouting are incompletely characterized, the ciliary axoneme protein Arl13b regulates scp guidance, which relies on non-cell autonomous Hedgehog signaling [459]. Changes in ARL13B expression cause abnormalities in growth cone dynamics and axonal tract development [458, 460]. Although axonal tract alterations of the dentato-thalamic tract in JS and JSRD have been described extensively, it remains to be determined whether earlier stages of CN development are also affected. Neuropathological studies have revealed abnormalities including dysplasia and hypoplasia of the Lat in JSRD [378, 461–463]. Interestingly, one patient with overt CN alterations exhibited drug-resistant epilepsy, a clinical sign that is seldom observed in JS patients [378].

### The Cerebellar Nuclei and Autism

Human functional magnetic resonance imaging studies have implicated the cerebellum in addiction [464–466], social cognition [467], and emotional processing [468]. In keeping with that evidence, cerebellar lesions or resections lead to various forms of cognitive impairment and abnormal social behavior [469]. Cerebellar abnormalities have also been linked to autism-like manifestations in genetically engineered mouse models [470–472] and to schizophrenia in humans [473].

Autism and autism spectrum disorder (ASD) are neurodevelopmental disorders characterized by deficits in communication, cognition, and behavior [474]. There is increasing evidence that many autism and ASD patients exhibit hypoplasia or other alterations of the cerebellum [470, 475–482] and defects in eye-blink conditioning [483]. Patients suffering from genetic or traumatic defects of the cerebellum may display changes in affective, behavioral, and cognitive abilities, including language difficulties, planning, and abstract conceptualizations [480–483, 485, 487].

A number of studies have reported changes in the CN [reviewed in 487, 488]. More recently, neuroimaging studies have described alterations involving the middle and superior cerebellar peduncles of ASD patients compared to healthy controls [489–491]. These results underline the potential impact of changes in the reciprocal connectivity between the cerebellum and neocortex in ASD pathogenesis, suggesting that they may be a consequence of structural, functional, or developmental changes of the CN themselves.

In keeping with the hypothesis that ASD is a connectivity disorder, the associations between cerebellar dysfunction and functional alterations of other areas of the brain can tentatively explain changes in sensory-motor control as well as language, social and emotional interaction, and cognition. Since the CN start forming earlier than the cerebellar cortex, they may be acting as an organizer in the ontogenesis of cerebellar cortical circuits. Disruption of the timing of CN development may impair their connectivity and cause dysfunction of the corresponding cortical targets. To shed light on the complexity of this neurodevelopmental disorder, it is essential to fully address the development of both local CN circuits and CN projections into thalamo-cortical relays [4].

### The Cerebellar Nuclei and Eating Disorders

Several neuroanatomical studies have shown that the cerebellum has direct and indirect bidirectional connections with the hypothalamus [493, 494]. The existence of cerebellar-hypothalamic circuits implicates the cerebellum in an integrated center for non-somatic visceral and homeostatic functions. Li *et al*. [495] demonstrated that cerebellar GABAergic and glutamatergic neurons of the Med modify the activity of the hypothalamic ventromedial nucleus, which in turn modulates feeding-related gastric signals, suggesting an important involvement for the cerebellar Med in feeding control. How this cerebellar control takes place from a molecular and physiological perspective remains an open research question of considerable importance.

In addition to the Med, the Lat has also been implicated in the regulation of satiety and the cerebellar components involved in regulating satiation were recently identified and functionally characterized in the Lat [5]. A subpopulation of glutamatergic neurons in the Lat, likely corresponding to Class-B glutamatergic projection neurons (Table 3), is activated by feeding or nutrient infusion into the intestine, and their specific activation substantially decreases food intake. These neurons project to the ventral tegmental area and increase basal levels of dopamine in the ventral striatum, thus attenuating the phasic dopamine response subsequent to food consumption. These observations define a satiation center that may represent a novel therapeutic target (e.g., via magnetic stimulation) for the management of compulsive eating disorder and subsequent obesity [5].

### The Cerebellar Nuclei and Depression

Patients diagnosed with major depressive disorder (MDD) experience at least one depressive episode that may involve both motor and cognitive symptoms [496]. Common cognitive signs and symptoms include difficulty concentrating or indecisiveness. While these MDD signs and symptoms are linked to functional changes in the prefrontal cortex and limbic system [497], several authors have described various abnormalities in the cerebellum of MDD patients, including a significantly smaller vermis [498].

Bipolar disorder is characterized by alternating periods of mania and depression, with manic episodes lasting at least a week and depressive symptoms appearing immediately afterwards [496]. Manic periods may involve euphoric moods, feelings of grandeur, hyperactivity, and impulsion, while depressive symptoms may consist of a lack of motivation, psychomotor agitation, or retardation [496]. The disorder is commonly a chronic lifelong condition. As is the case for MDD, studies conducted in patients with bipolar disorder have shown evidence of cerebellar involvement, with decreased cerebellar volume and cerebellar atrophy [499–501].

While most analyses of the cerebellum in mood disorders have focused on the cerebellar cortex, CN neurons projecting to the ventral tegmental area are known to play a key role in the development of chronic stress-induced behavioral alterations in mice. In one study, chronic chemogenetic activation of PCs in crus I was found to suppress the expression of the immediate early gene *c-Fos* in the Lat and to attenuate the immobility response in the tail suspension or forced swimming test, triggered by chronic stress. In the same study, circuit mapping and electrophysiology experiments revealed a connection from crus I of the cerebellar hemispheres to the ventral tegmental area, mediated by the Lat. Moreover, depression-like behavior was reduced by chronically inhibiting Lat neurons that project to the ventral tegmental area, while their sustained activation alone triggered depression-like behaviors [502]. These results indicate that functional deregulation of the Lat neurons projecting to the VTA is a key factor in the development of depression-like manifestations and may affect general processing of rewards [503]. Such neurons may be an effective target for the prevention of depressive disorder in humans [273, 502].

### The Cerebellar Nuclei and Other Disorders

SCA, FA, and JS are all examples of genetic disorders that have long been known to involve the cerebellum. However, the cerebellum’s role in other neurodegenerative diseases, such as Parkinson’s and Alzheimer’s diseases, was relatively unexamined until the past decade. Recent studies of Parkinson’s disease patients utilizing functional and structural MRI, PET imaging or deep brain stimulation have made it possible to attempt to explain the occurrence of tremor in this disorder, and its relative contribution to the clinical picture that also involves bradykinesia and rigidity. In Parkinson’s disease, tremor severity correlates poorly with other motor symptoms [389]. Moreover, tremor can in some cases affect the side of the body opposite to the one that is more affected by bradykinesia and rigidity [504]. Finally, tremor responds less well to dopaminergic treatment than bradykinesia and rigidity [390]. Many lines of evidence support the notion that tremor in Parkinson’s disease has an important cerebellar component [reviewed in 391]. While most studies point to the involvement of the cerebellar cortex (particularly lobules IV and V), PET results have also highlighted a tremor-related network including the Lat [391].

In one study, patients affected by another neurodegenerative disorder, Alzheimer’s disease, which is rarely linked to the cerebellum, were found to exhibit significantly higher levels of cell cycle markers and DNA damage response proteins in the Lat [359]. High levels of these molecular markers correlate with the less characterized cerebellar signs of Alzheimer’s disease, including deficits in speech, language, and motor planning [359].

Recently, the electrophysiological activity of the cerebellum was investigated in the APPswe/PSEN1dE9 mouse model of Alzheimer's disease, revealing signs of electrophysiological alterations in both PCs and CN neurons. These results highlight the importance of changes in cerebellar output firing, possibly affecting the function of cerebellar target circuits at subcortical and cortical locations [505]. This and other findings suggest that in addition to coordinating the motor functions of the cerebellum, the CN may also play important roles in the cerebellum’s higher order functions, including cognition and emotion [e.g., 509].

### The Promise of Deep Brain Stimulation of the Cerebellar Nuclei as a Therapeutic Approach

The compact and restricted topography of CN warrants efforts aimed at targeting their function to modulate the progression of cerebellar disorders. As an example, the potential benefit of deep brain stimulation of the interposed and lateral nuclei in the management of dystonia and stroke, respectively, was exhaustively discussed in a recent review [506]. Mouse models of dystonia obtained by conditionally inactivating the glutamatergic output of the inferior olive to the cerebellum [507] were successfully approached by electrically stimulating the interposed nuclei, which project their output mainly to the red nucleus. In turn, the red nucleus is known to be part of a descending pathway that activates the inferior olive [507]. Likewise, a rat model of stroke was treated by deep-brain [508] and optogenetic [442] stimulation of the Lat, leading in both cases to encouraging results. These preclinical experiments are being replicated in stroke patients. In summary, deep brain stimulation of the CN holds promise in the management of severe neurologic disorders, although the underlying mechanism of action awaits clarification.

## Conclusions and Future Directions

In this review, we have provided a comprehensive overview of the morphology, cytology, anatomy, physiology, development, evolution, and clinical relevance of the CN. As such, we hope to have convinced readers that these underappreciated nuclei form the center of the cerebellum not only in a literal sense, but also figuratively, as they play a pivotal role in cerebellar function. While reviewing the wealth of knowledge available, we became increasingly conscious of numerous unanswered questions. In this last section, we wish to highlight the most critically needed information for understanding the entire cerebellum’s role in brain function.

From anatomical studies, it is obvious that the CN lie at the heart of the modular organization of the olivo-cortico-nuclear system. Although evidence has emerged that the individual nuclei (i.e., Med, IntP, IntA, and Lat) are subdivided into several subnuclei or subdivisions, each forming their own functional unit, the level to which the nuclei can be coherently subdivided to form these modules is not known. This question is especially pertinent when the level of compartmentalization of the cerebellar cortex is likely to hugely exceed that of the number of presently recognized number of CN subdivisions [72, 123, 129]. Hence, information is critically needed to derive a better understanding of the organization of the micromodular aspects of the corticonuclear projections as well as the intra-CN synaptic connectivity. In this respect, it would be required to see how this organization deals with the currently established five canonical CN cell types. At what level is the modular organization present and when do the modules start to show overlapping or diverging characteristics?

Of course, this question also relates to how the physiological modules exert their function. Although we have not discussed the cerebellar cortical processing of MF and CF input, it is relevant to understand how the result of this processing coincides with the direct input by precerebellar systems to the nuclei and ultimately results in a coordinated output action to the rest of the brain. At present, the anatomical and physiological data are not straightforward to evaluate. One aspect that needs considerable clarification is how the output of individual CN neurons is distributed to the rest of the brain. Although specific parts of the CN are claimed to target selected areas of the brainstem (Fujita, [20]), other projections diverge, for example, to the spinal cord and thalamus, suggesting that divergence and simultaneously impacting multiple systems with the same information is the rule rather than the exception. Recently, it has been shown that even the axons of the only CN cell type with a presumptive designated target, the nucleo-olivary cells, may collateralize to several other, non-olivary, areas in the brainstem (Judd, [151]). Apart from divergence, convergence also seems to be a rule. Indeed, the output of different modules, for example by way of their CN projections to the thalamus, also seems to impact identical regions of the cerebral cortex (Aoki, [170]). As such, the diverging and converging characteristics of the clearly modular basic organization of the olivo-cortico-nuclear modules require additional attention.

A full understanding of these connections is only likely to occur when the developmental and evolutionary research lines enable a fusion of ontogeny, phylogeny, anatomy, and physiology. Some of the pressing questions in the realms of development and evolution are noted below.

From a developmental point of view, we have learned much about the glutamatergic CN neurons by using Atoh1-tagged mice. However, the GABAergic part of this story is at best still incomplete and the use of similar lines of tagged PTF1A mice would be invaluable to address the question if the development of the SOX14-defined nucleo-olivary projection neurons is dependent on PTF1A in particular and to examine the relationship of PTF1A to the inhibitory populations of CN neurons in general.

Flow sorting and tagging single cells/nuclei for sequencing has opened an exciting new territory in the molecular and cellular analyses of CN development. Some of the following important questions should be answered. What is the temporal signal that patterns successive cohorts of CN in the RL that are destined to different nuclei? How discrete are these temporal cohorts? Which cues regulate the integration of glutamatergic and GABAergic cells in nucleogenesis? How and when do the “rule-breaking” RL-derived, nucleus-specific, glycinergic neurons of the Med developmentally diverge from the excitatory lineage, and when do they establish their glycinergic phenotype?

One outstanding question that crosses both developmental and evolutionary domains is why the CN neurons, in contrast to the cerebellar cortex, aggregate into discrete structures in the first place. Explanations might be sought into providing confined terminal fields of CN interneurons or CN afferents, or bundling of the CN efferents. Alternatively, the subnuclear aggregates of the individual CN may prove to be an anatomical and developmental consequence of CN mediolateral duplication through evolution (“Evolutionary Origins of the Cerebellar Nuclei”).

In this evolutionary model, the addition of each new cluster would represent a specific functional adaptation to a changing or expanding role of the cerebellum in the CNS. Each duplicated population of cells would likely correspond to a distinct developmental window and a discrete anatomical identity. These duplications would provide more CN but not necessarily novel functions and may relate to the overlap in adult connectivity between different nuclei. A modern terminology from these perspectives would talk about Med, Int, and Lat nuclear and subnuclear clusters—with the evolutionary relationships of the extant CN inferred from scRNAseq data. How these clusters are represented and map to the full range of vertebrate cerebella will be a fascinating story.

It is clear that different branches of the evolutionary tree display their own CN innovations, such as the Lat and IntA of mammals and a related CN region, IntX, that is exclusive to birds [35]. Will genetic analysis reveal further diversification in, for example, cetaceans where the interpositus nuclei undergo a massive expansion in relative size [25], reminiscent of the Lat in the great apes and humans? While powerful, single-cell genomic approaches will go a long way to answering these questions, they will not be sufficient to unravel the functional significance of evolutionary adaptations. Here the power of single-cell analysis will need to be combined with an understanding of afferent and efferent projections and the novel circuits and modules in which the evolutionary diversity of the CN participates. Indeed, it would be helpful to gain more knowledge concerning the evolutionary origins of the CN. For example, are the teleost eurydendroid cells a distributed population of CN cells that collectively function as a single CN? Does the CN in the shark rostral hindbrain comprise the same basic repertoire of neuronal types found in chicks, mice, and humans, and if so, which?

Finally, despite the central role played by CN neurons in cerebellar function and evolution, there are still many open questions regarding their contribution to human cerebellar abnormalities. While data supporting a causal involvement of CN in human disease remain sketchy in most cases, mounting evidence suggests a correlation between several neurodevelopmental or neurodegenerative disorders and multiform alterations of the CN. Unfortunately, after 60 years of in vivo studies centered on spontaneous and genetically engineered mice, while there are many genetic mutations that affect the cerebellum [e.g., 510], not one CN-specific mutant has been described. This suggests at least three explanations: (1) the impairment of CN function in all lines inspected to date may only cause incremental or minor effects on the overall phenotype; (2) gene mutations causing a major disruption of CN function leading to prenatal death; or (3) the loss of the CN in development may result in major secondary reductions in the cerebellar cortex and hence be ascribed to a primary cortical deficit. We believe that functional studies, as opposed to mere correlative ones, are required, focusing on the contributions of CN to human neurologic and neurodevelopmental disease.

To this end, new tools are critically needed. Recent single-cell unsupervised transcriptome analyses (see above) have cast light on the transcriptional landscape of the developing and adult CN, leading to the identification of CN-specific markers as well as factors selectively expressed by specific CN neuron subpopulations. This should foster the generation of CN-specific Cre lines for conditional mutagenesis, or the design of new strategies for intersectional transgenesis. Indeed, by selectively inactivating or misexpressing genes in the embryonic, postnatal, or adult CN it will become possible to gauge their relative contribution to human disorders featuring potential CN pathology or dysfunction. This information is highly relevant in that it may reveal novel pathogenetic components of nervous system disorders and, in selected cases, may identify the CN as a target for therapeutic intervention, including deep brain stimulation, gene therapy, and epigenetic editing.

In summary, by reviewing existing knowledge on the CN, we are convinced that dealing with these questions will go a long way towards understanding the function of the CN and will allow a nucleocentric understanding of the cerebellum as a whole. We are excited to continue this journey and invite the reader to join us at the heart of the cerebellum—the cerebellar nuclei.

## Data Availability

Not applicable.

## Abbreviations

ASD: Autism spectrum disorder
CF: Climbing fiber
CN: Cerebellar nuclei
CNS: Central nervous system
FA: Friedreich’s ataxia
Int: Interposed nuclei
IntA: Anterior interposed nucleus
IntP: Posterior interposed nucleus
IO: Inferior olive
JS: Joubert syndrome
JSRD: JS-related disorder
Lat: Lateral cerebellar nucleus
Med: Medial cerebellar nucleus
MedDL: Dorsolateral protuberance of the Med
MF: Mossy fiber
NTZ: Nuclear transitory zone
PC: Purkinje cell
RL: Rhombic lip
SCA: Spinocerebellar ataxia
scp: Superior cerebellar peduncle
SPS: Subpial stream
VMN: Ventromedial nucleus of the hypothalamus
VZ: Ventricular zone
